# BIG1 is required for the survival of deep layer neurons, neuronal polarity, and the formation of axonal tracts between the thalamus and neocortex in developing brain

**DOI:** 10.1371/journal.pone.0175888

**Published:** 2017-04-17

**Authors:** Jia-Jie Teoh, Tomohiko Iwano, Masataka Kunii, Nur Atik, Erda Avriyanti, Shin-ichiro Yoshimura, Kenta Moriwaki, Akihiro Harada

**Affiliations:** 1 Department of Cell Biology, Graduate School of Medicine, Osaka University, Suita, Osaka, Japan; 2 Department of Anatomy and Cell Biology, Interdisciplinary Graduate School of Medicine and Engineering, University of Yamanashi, Chuo, Yamanashi, Japan; 3 Department of Anatomy and Cell Biology, Faculty of Medicine, Padjadjaran University, Bandung, Indonesia; 4 Department of Dermatology and Venereology, Faculty of Medicine, Padjadjaran University, Bandung, Indonesia; Hokkaido Daigaku, JAPAN

## Abstract

BIG1, an activator protein of the small GTPase, Arf, and encoded by the *Arfgef1* gene, is one of candidate genes for epileptic encephalopathy. To know the involvement of BIG1 in epileptic encephalopathy, we analyzed BIG1-deficient mice and found that BIG1 regulates neurite outgrowth and brain development *in vitro* and *in vivo*. The loss of BIG1 decreased the size of the neocortex and hippocampus. In BIG1-deficient mice, the neuronal progenitor cells (NPCs) and the interneurons were unaffected. However, Tbr1^+^ and Ctip2^+^ deep layer (DL) neurons showed spatial-temporal dependent apoptosis. This apoptosis gradually progressed from the piriform cortex (PIR), peaked in the neocortex, and then progressed into the hippocampus from embryonic day 13.5 (E13.5) to E17.5. The upper layer (UL) and DL order in the neocortex was maintained in BIG1-deficient mice, but the excitatory neurons tended to accumulate before their destination layers. Further pulse-chase migration assay showed that the migration defect was non-cell autonomous and secondary to the progression of apoptosis into the BIG1-deficient neocortex after E15.5. In BIG1-deficient mice, we observed an ectopic projection of corticothalamic axons from the primary somatosensory cortex (S1) into the dorsal lateral geniculate nucleus (dLGN). The thalamocortical axons were unable to cross the diencephalon–telencephalon boundary (DTB). *In vitro*, BIG1-deficient neurons showed a delay in neuronal polarization. BIG1-deficient neurons were also hypersensitive to low dose glutamate (5 μM), and died via apoptosis. This study showed the role of BIG1 in the survival of DL neurons in developing embryonic brain and in the generation of neuronal polarity.

## Introduction

The neocortex, part of the cerebral cortex, is the evolutionary newest part of the brain and is responsible for most of the higher brain functions including perception, language and decision-making [1]. This results from the orderly migration, correct localization and accurate integration of various types of neurons, including excitatory neurons and inhibitory interneurons into functional neuronal networks, beginning during embryonic brain development [2]. Unlike the inhibitory interneurons that arise from the progenitor pool in the subpallium, where the ganglionic eminence (GE) is located [3], the excitatory neurons arise from the neuronal progenitor cells (NPCs) lining the ventricular zone and the subventricular zone (VZ/SVZ) in the pallium [4]. As they mature, these neurons then migrate radially into various layers of the cortical plate in the neocortex [5, 6]. Among these layers, the UL excitatory neurons project axons into the contralateral brain hemisphere while DL excitatory neurons extend their axons into the ipsilateral thalamus [7].

To date, five families of ADP-ribosylation-factor -specific guanine nucleotide exchange factors (ARF-GEFs), which have a conserved catalytic sec7 domain, have been identified [8]. Unlike the others, the GBF/BIG family is characterized based on both large molecular size and sensitivity to Brefeldin A (BFA) [9, 10]. BFA-inhibited guanine-nucleotide-exchange proteins-1 (BIG1), encoded by the *Arfgef1* gene, is one of the largest ARF-GEFs found in mammals, and conserved homologues of BIG1 have been found in all eukaryotes studied [8, 11]. This 200 kDa protein was initially identified as a component of a 670 kDa cytosolic macrocomplex purified from the bovine brain [12]. Similar to BIG2 and GBF1, which belongs to the same family, BIG1 contains a 200 amino acids BFA-sensitive Sec7 domain that preferentially activates Class I ARFs, catalyzing the conversion of ARF-GDP to ARF-GTP, thereby initiating the formation of intracellular vesicles [13, 14, 15].

Among the large ARF-GEFs, BIG2 had been shown to be involved in neuronal migration in mammalian brain [16]. Similarly, BIG1 has been shown to regulate neurite development *in vitro* [17]. BIG1 localizes to the Golgi complex, and perturbation of BIG1 with BFA affects the function of the Golgi complex [14, 18]. Meanwhile, in a recent exome-sequencing screening study in human, mutation of *Arfgef1*, was identified as a candidate of the most damaging genetic causes for infantile epileptic encephalopathy [19]. While BIG1 appears to function in brain development and pathogenesis of epilepsy, there have been no *in vivo* studies of BIG1 function in mammalian brain. Here, we elucidate the role of BIG1 in the survival and the connectivity of DL neurons which would help to explain the pathogenicity of epileptic encephalopathy due to mutation of the *Arfgef1* gene.

## Materials and methods

### Ethics statement

All animals were bred and housed at the Institute of Animal Experimental Research in Osaka University. All animal experiments conformed to the institutional guidelines and were approved by the Animal Care and Experimentation Committee of Osaka University (Approval No.21-045-0). All animals used in the study were euthanized with an overdosed by isoflurane prior to transcardial perfusion or tissue dissection. All efforts were made to minimize suffering.

### Antibodies

The following primary antibodies were used: anti-BIG1 N-terminal (Santa Cruz, sc-376790), anti-BIG1 C-terminal (Bethyl, A300-998A), anti-Lamin-B (Santa Cruz, sc-6216) anti-Pax6 (Covance, PRB-278P), anti-cleaved-Caspase-3 (Cell Signaling, #9661), anti-Calbindin (Santa Cruz, sc-7691), anti-Prox1 (R&D Systems, AF2727), anti-Ctip2 (Abcam, ab18465, gift from Dr. Makoto Sato, Osaka University), anti-Tbr1 (Chemicon, AB9616; Santa Cruz, sc-15607), anti-Cux1 (Santa Cruz, sc-13024), anti-BrdU (BD Pharmingen, 555627), anti-Map1b (clone 1B6, gift from Dr. Reiko Harada, Takarazuka University of Medical and Health Care) [20], anti-Tau1 (Chemicon, LV1383492), anti-Map2 (Cell Signaling, #4542), anti-Tuj1 (Covance, MMS-435P) and anti-Golgin97 (Gift from Dr. Nobuhiro Nakamura, Kyoto Sangyo University) [21]. The following secondary antibodies were used: HRP-conjugated goat anti-mouse (ICN Pharmaceuticals, 59296), calf anti-rabbit (Rockland, 200–4135) or calf anti-goat (Rockland, 200–1135), Alexa-Fluor-488-labeled or Alexa-Fluor-594-labeled donkey anti-mouse (Invitrogen, A21202; Molecular Probes, A21203), donkey anti-rabbit (Invitrogen, A21206; Molecular Probes, A21207), donkey anti-rat (Molecular Probes, A21208) and donkey anti-goat (Molecular Probes, A11055).

### Generation of *Arfgef1*^*geo/geo*^ mice

The mice heterozygous for the *Arfgef1* gene-trapped allele (*Arfgef1*^*geo/+*^) were generated using a mouse ES cell line, CSH465 (Bay Genomics). Mice at two to six-months-old were used for mating. *Arfgef1*^*geo/+*^ mice were intercrossed to obtain mice homozygous for the *Arfgef1* trapped allele (*Arfgef1*^*geo/geo*^), as well as *Arfgef1*^*geo/+*^ and wild-type *(Arfgef1*^*+/+*^) progeny. The progeny had a mixed 129/SvJ and C57BL/6J genetic background. All mice were kept under a 12-hr light-dark cycle with unlimited access to water and food. The progeny embryos, regardless of sexes, were used in this study.

### RT-PCR

Total RNA was collected from *Arfgef1*^*geo/geo*^ and *Arfgef1*^*+/+*^ brains at E17.5. Reverse transcription was performed using Prime-Script Reverse transcriptase (Takara, 2680A) according to the manufacturer's protocol. PCR was then performed using primers flanking BIG1 cDNA corresponding to the sec7 domain (exons 14–18), exons 32–35 and exons 36–38. The PCR products were subjected to electrophoresis on agarose gels.

### Western blot

Brain lysates were collected using the trichloroacetic acid precipitation method. Western blotting was performed as previously described [22]. Briefly, the lysates were separated by SDS-PAGE and transferred to PVDF membranes (Millipore, IPVH00010). The membranes were incubated with primary antibodies overnight at 4°C, followed by incubation with the appropriate HRP-conjugated secondary antibody for 1 hr at room temperature. Samples were washed three times in PBS with 0.05% Tween-20 (PBST) after each step. Signals were developed using a chemiluminescent HRP substrate (Millipore, WBKLS0500) and visualized using X-ray film (Fuji Medical, 47410 26617).

### Histological processing and TUNEL assay

The E17.5 embryonic brains were collected and fixed in 4% (w/v) paraformaldehyde (PFA) in 0.1 M phosphate buffer (PB) at pH 7.4 overnight at 4°C. After dehydration, the brains were then embedded in paraffin and sectioned with a microtome (Leica, RM2145) at 6 μm. Every tenth section was mounted on a glass slide (Matsunami Glass, FF-003) and subjected to hematoxylin and eosin (HE) staining as described previously [23]. The TUNEL assay was performed using the brain paraffin sections with a colorimetric TUNEL assay kit (Promega, G7132) according to manufacturer's protocol.

### Immunofluorescence

For immunofluorescence of brain sections, embryonic brains were collected and fixed with 4% (w/v) PFA in 0.1 M PB at pH 7.4. Fixation time was six hrs for E13.5 and E15.5 brains and overnight for E17.5 brains. Brains were cryoprotected by soaking in step-wise increments of 10%, 15% and 20% sucrose in 0.1 M PB at pH 7.4 at 4°C. The tissues were then embedded in O.C.T. compound (Sakura Finetek, 4583) and frozen in isopentane chilled in liquid nitrogen. The cryosections were sliced at 12 μm using a cryostat (Leica Biosystems, CM3050S), mounted on MAS-GP coated glass slides (Matsunami Glass, S9905), dried, and stored at -80°C until immunofluorescence staining. Structure- and position-matched sections of *Arfgef1*^*geo/geo*^ and control brains were selected for staining and comparison. For all nuclear proteins, an antigen retrieval step was performed using HistoVT One (Nacalai Tesque, 06380–05) according to the manufacturer’s recommended protocol. Staining of cryosections began with incubation with blocking buffer (5% normal donkey serum and 0.2% Triton X-100 in PBS) at room temperature for 30 min, followed by incubation with primary antibodies in the staining buffer (5% normal donkey serum and 0.01% Triton X-100 in PBS) at 37°C for 1 hr or at 4°C overnight and then incubation with Alexa dye-conjugated secondary antibodies in staining buffer at room temperature for 1 hr in the presence of DAPI (Roche, 236276). Samples were washed three times in PBS after each step.

For immunofluorescence, neurons were fixed for 10 min with 4% (w/v) PFA in 0.1 M PB at pH 7.4. The staining began with a 20 min incubation in blocking buffer (5% normal donkey serum and 0.2% saponin in PBS), followed by incubation with primary antibodies in staining buffer (5% normal donkey serum and 0.01% saponin in PBS) at 37°C for 1 hr and then incubation with DAPI and Alexa dye-conjugated secondary antibodies in staining buffer at room temperature for 1 hr. Samples were washed three times in PBS after each step. The samples were mounted with a cover glass using Mowiol mounting medium containing 1,4-diazabicyclo-[2,2,2]-octane (DABCO) as an anti-fade agent.

### Image collection, processing and quantifications

All images were collected using a C-5060 camera (Olympus) mounted on a CKX41 inverted microscope (Olympus), DP80 camera (Olympus) mounted on a BX61 fluorescence microscope (Olympus), BZ-9000 all-in-one fluorescence microscope (Keyence) or FV-1000D confocal microscope (Olympus). Images were processed using Adobe Photoshop version 8.0.1 (Adobe Systems). Quantification of cell numbers was performed using Adobe Photoshop. For Pax6^+^, Ki67^+^ or BrdU^+^ cells, 200 μm of cortical width starting from the lateral ventricle edge in E13.5, E15.5 and E17.5 brains was selected for calculation. The marker-positive cells are presented as the percentage of total DAPI^+^ cells within the selected area. For Tbr1^+^ and Cux1^+^ neurons, 200 μm cortical areas in E17.5 brains were selected as described above and separated into 10 bins from the ventricular zone (VZ) to the marginal zone (MZ). The distributions of marker-positive cells in each bin were calculated and are presented as the percentage of DAPI^+^ cells within respective bin. Quantification of colocalized Caspase-3 and Tbr1 or Ctip2 signals in *Arfgef1*^*geo/geo*^ brains was conducted according to spatial-temporal distribution of apoptotic cells. At E13.5, 200 μm^2^ at piriform cortex was selected for quantification. At E15.5 and E17.5, 200 μm of cortical width in somatosensory cortex and retrosplenial cortex was selected for quantification respectively. The thickness of corpus callosum (CC) was quantified as the percentage relative to the brain height or width. Quantification of hippocampal neurons properties was performed using Fiji software [24] with the NeuronJ plugin [25].

### Pulse-chase neuronal migration assay

Timed-pregnant dams were intraperitoneally injected with 50 mg/kg bromodeoxyuridine (BrdU) (Sigma, B9285) at E12.5. The embryonic brains were collected after 72 hrs at E15.5, processed, immunostained with anti-BrdU antibody and counterstained with DAPI. The neocortex was divided into VZ/SVZ, IZ and CP. The percentages of BrdU^+^ cells relative to DAPI^+^ cells in each division were scored.

### Dye tracing

The E17.5 brains were collected and fixed as described above. After replacing the fixative with fresh 4% PFA in 0.1 M PB, the samples were kept at 4°C until dye tracing. Samples stored less than 2 months were used. For corticothalamic or contralateral commissural axon tracing, a small Neuro-DiI (Promokine, PK-CA707-60016) crystal was placed in the primary somatosensory cortex (S1). For thalamocortical axon tracing, the crystal was placed in the thalamus. To accelerate diffusion, the samples were incubated at 37°C. The samples were then embedded in 4% agarose (Sigma, A6013), and sections at 100 μm thickness with a vibratome (Leica, VT1000S). Free-floating sections were counterstained with DAPI and then mounted on a glass slide with glycerol for examination.

### Culture of primary hippocampal neurons

Primary hippocampal neurons were collected according to a previously published method [26]. Briefly, hippocampi were dissected from E17.5 embryos, incubated with dissociation solution containing papain (Sigma, P5306), 1% BSA (Sigma, A7906), DNase (Sigma, DN25) and glucose (Wako, 049–31165) in PBS (Wako, 167–14491) and dissociated using trituration. A total of 1 x 10^5^ cells were plated in each 35 mm culture dish coated with poly-D-lysine (Sigma, P7280) and laminin (Sigma, L2020). The plating medium, which contained Neurobasal medium (Gibco, 21103–049), Glutamax (Gibco, 35050–061) and 10% fetal bovine serum (Equitech-Bio, SFBM30-2501) was replaced after 4 hrs with normal culture medium containing Neurobasal medium supplemented with B27 (Gibco, 17504–044) and Glutamax. The cultures were maintained at 37°C and 5% CO_2_ in a humidified incubator with 50% medium change every three days until the experiment.

### Glutamate and GABA treatment

The primary hippocampal neurons were collected as described above. The glutamate stock solution was prepared as 10 mM of glutamic acid diluted in 10 mM NaOH. The GABA stock solution was prepared as 10 mM. Both were stored at -20°C until use. At day *in vitro* (DIV) 15, the neurons were treated for 5 min with glutamate in 1 ml control salt solution (CSS), containing 156 mM NaCl, 3 mM KCl, 2 mM MgSO_4_, 1.25 mM KH_2_PO_4_, 2 mM CaCl_2_, 10 mM glucose and 10 mM HEPES, adjusted to pH 7.4 with 10 N NaOH, with three washes in CSS before and after glutamate treatment. The samples were then returned to the humidified incubator at 37°C and 5% CO_2_ in normal culture medium. GABA treatments were performed at DIV 4 as describe above. Cells were examined using an inverted microscope after 1, 3, 6 and 12 hrs. Samples at 30 min after the glutamate treatments were immunostained for activated Caspase-3 and Tuj1.

## Results

### Genetic analysis of BIG1 gene-trap mice

**Fig 1:**
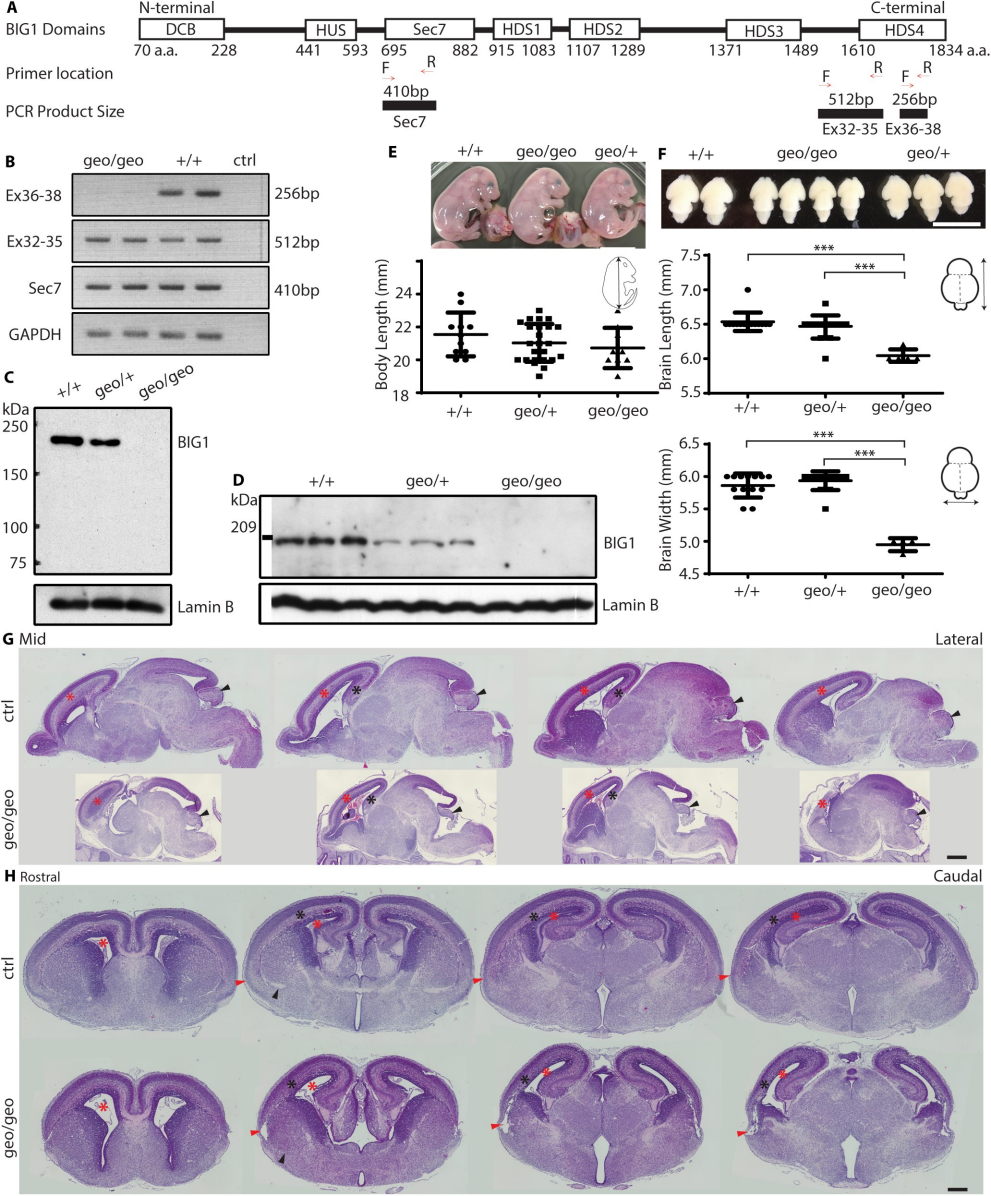
Genetic and morphological analyses of BIG1 gene-trap mice. (A) The forward (F) and reverse (R) PCR primers flanking the Sec7 domain, exons 32–35 and exons 36–38 of BIG1 cDNA were used for PCR. The expected PCR product sizes are shown. The cDNA regions corresponding to respective domains in BIG1 protein are shown and labeled. (B) Representative electrophoresis gel images of PCR products (n = 2 per group). (C-D) The western blot was performed using E17.5 brain lysate and detected using antibody targeting BIG1 based on an (C) N-terminal epitope (n = 3 brains per group) or (D) C-terminal epitope, using Lamin B as loading control. (E) The length of E17.5 embryos among the three genotypes is statistically similar. *Arfgef 1*^*+/+*^ vs *Arfgef1*^*geo/geo*^, p = 0.08; *Arfgef1*^*geo/+*^ vs *Arfgef1*^*geo/geo*^, p = 0.14. Scale bars, 10 mm. *Arfgef 1*^*+/+*^, n = 11; *Arfgef1*^*geo/+*^, n = 24; *Arfgef1*^*geo/geo*^, n = 9. (F) At E17.5, the length and width of brains in *Arfgef1*^*geo/geo*^ mice are statistically smaller than *Arfgef1*^*geo/+*^ and *Arfgef 1*^*+/+*^ mice. Length, *Arfgef 1*^*+/+*^ vs *Arfgef1*^*geo/geo*^, ***p = 9.20E-07; *Arfgef1*^*geo/+*^ vs *Arfgef1*^*geo/geo*^, ***p = 4.07E-06. Width, *Arfgef 1*^*+/+*^ vs *Arfgef1*^*geo/geo*^, ***p = 2.15E-10; *Arfgef1*^*geo/+*^ vs *Arfgef1*^*geo/geo*^, ***p = 1.36E-10. Scale bars, 5 mm. *Arfgef 1*^*+/+*^, n = 13; *Arfgef1*^*geo/+*^, n = 14; *Arfgef1*^*geo/geo*^, n = 4. (G) HE-stained sagittal sections shows differences in the neocortex (red asterisks), hippocampus (black asterisks) and cerebellum (black arrowheads) between *Arfgef1*^*geo/geo*^ brains and controls. Scale bars, 500 μm. n = 3 per group. (H) HE-stained coronal sections showing differences in the neocortex (black asterisks), lateral ventricles (red asterisks), piriform cortex (red arrowheads) and external capsule (black arrowheads). Scale bars, 500 μm. n = 3 per group. For all quantifications, Student’s T-test was used for comparisons between groups. Data are shown as the mean ± SD.

We generated BIG1-deficient mice from ES cells that contain a gene-trap cassette with β-geo in the *Arfgef1* locus. The gene-trap cassette was inserted between exon 35 and exon 36, corresponding to the C-terminus of BIG1. This may result in a truncated functional protein. Therefore, we checked the amount of BIG1 mRNA using three pairs of primers, flanking the catalytic sec7 domain (exons 14–18), exons 32–35 and exons 36–38 (Fig 1A). We obtained PCR products from the former two primer pairs, suggesting that the truncated BIG1 mRNA was present in *Arfgef1*^*geo/geo*^ embryos (Fig 1B). However, we were unable to detect BIG1 protein in *Arfgef1*^*geo/geo*^ brains using BIG1 antibody against an N-terminal epitope (Fig 1C) or a C-terminal epitope (Fig 1D), confirming the complete absence of BIG1. Thus, we consider *Arfgef1*^*geo/geo*^ mice to be BIG1-deficient mice.

The genotypes of the progeny followed a normal Mendelian frequency (*Arfgef1*^*+/+*^, 22.5%; *Arfgef1*^*geo/+*^, 56.9%; *Arfgef1*^*geo/geo*^, 20.6%; n = 218). The *Arfgef1*^*geo/+*^ mice were viable and fertile without observable defects compared to *Arfgef1*^*+/+*^ littermates. *Arfgef1*^*geo/geo*^ pups died within one day after birth. Thus, we were unable to access their neurological phenotype in detail. However, their empty stomachs suggested the inability to feed on their own. Based on the early postnatal death of BIG1-deficient mice, BIG1 is likely to participate in embryonic development.

### Morphological defects of BIG1 homozygous gene-trap mice

At E17.5, the body sizes of *Arfgef1*^*geo/geo*^ embryos were slightly smaller than *Arfgef1*^*+/+*^, *Arfgef1*^*geo/+*^embryos but not statistically significant (Fig 1E). However, the length and width of the brains in *Arfgef1*^*geo/geo*^ embryos were significantly smaller than that in their *Arfgef1*^*+/+*^ or *Arfgef1*^*geo/+*^ littermates (Fig 1F). As sections from *Arfgef1*^*+/+*^and *Arfgef1*^*geo/+*^ brains showed no histological differences by HE staining, results from *Arfgef1*^*+/+*^and *Arfgef1*^*geo/+*^ were treated as controls. The brain of *Arfgef1*^*geo/geo*^ mice were smaller in the hippocampus (black asterisks), neocortex (red asterisks) and cerebellum (black arrowheads) (Fig 1G). When the coronal sections were examined, the *Arfgef1*^*geo/geo*^ neocortex was found to be thinner (Fig 1H). This reduction in size was accompanied by the enlargement of the lateral ventricles (red asterisks) (Fig 1H). Furthermore, the *Arfgef1*^*geo/geo*^ brains were also found to have a complete loss of the anterior commissure (black arrowheads) (Fig 1H).

### Loss of BIG1 did not change the number of NPCs

**Fig 2:**
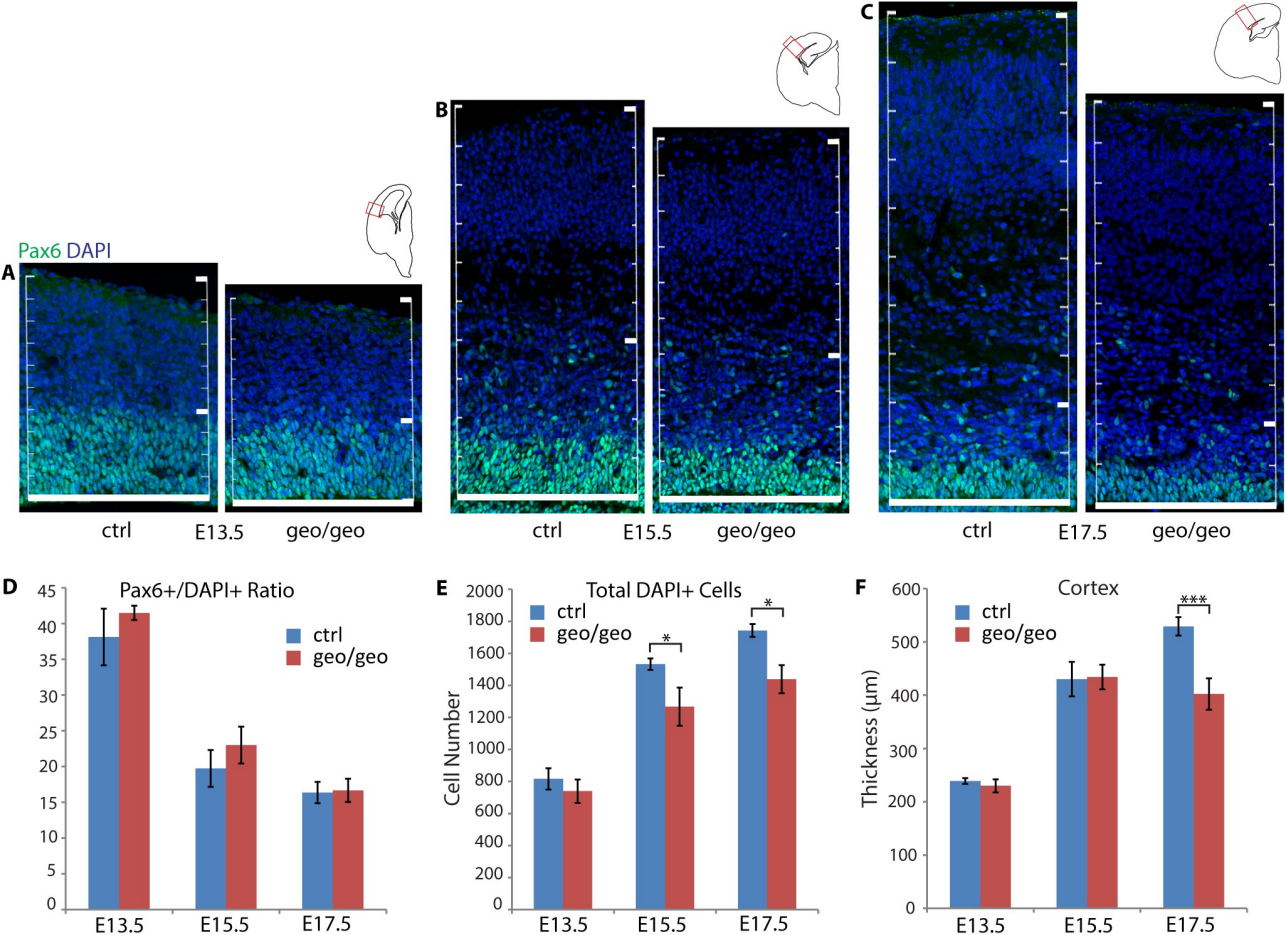
Loss of BIG1 does not change the proportion of NPCs. (A-C), Comparison of the numbers of Pax6^+^ and DAPI^+^ cells between *Arfgef1*^*geo/geo*^ and control embryos at E13.5 (A), E15.5 (B) and E17.5 (C). The numbers of cells from the edge of the lateral ventricle within 200 μm cortical width were counted. Scale bars, 200 μm. n = 3 per group. (D) The ratio of Pax6^+^ NPCs and DAPI^+^ cells did not have significant difference at E13.5 (p = 0.09), E15.5 (p = 0.10), or E17.5 (p = 0.41). n = 3 per group. (E) The total number of cells in *Arfgef1*^*geo/geo*^ neocortex is reduced at E15.5 (*p = 0.03) and E17.5 (*p = 0.01) but not E13.5 (p = 0.09). n = 3 per group. (F) The thickness of *Arfgef1*^*geo/geo*^ neocortex is reduced at E17.5 (***p = 6.10E-06) but not at E13.5 (p = 0.09) and E15.5 (p = 0.41). n = 3 per group. Student’s T-test was used for comparisons between groups. Data are shown as the mean ± SD. n = 3 per group for each time point.

To assess whether defects in the *Arfgef1*^*geo/geo*^ neocortex resulted from abnormalities in the NPCs, we first evaluated the ratio of Pax6^+^ NPCs in total DAPI cells. At E13.5 (Fig 2A), E15.5 (Fig 2B) and E17.5 (Fig 2C), there were no statistical differences in the ratios of Pax6^+^ NPCs (Fig 2D) between *Arfgef1*^*geo/geo*^ and control mice. This indicated that the ratio of Pax6^+^ NPCs was maintained to a percentage similar to control in *Arfgef1*^*geo/geo*^ neocortex throughout embryonic development. On the other hand, at E15.5 (Fig 2B) and E17.5 (Fig 2C), there were significantly fewer total DAPI^+^ cells per unit of cortical width in *Arfgef1*^*geo/geo*^ than in control mice (Fig 2E). Combining with the result that the cortex thickness was significantly thinner in *Arfgef1*^*geo/geo*^ brain only at E17.5 (Fig 2F), this suggested that the reduction in postmitotic neurons, but not Pax6^+^ NPCs, were most likely to contribute to the reduction in cortical thickness.

### Loss of BIG1 caused neuronal apoptosis

**Fig 3:**
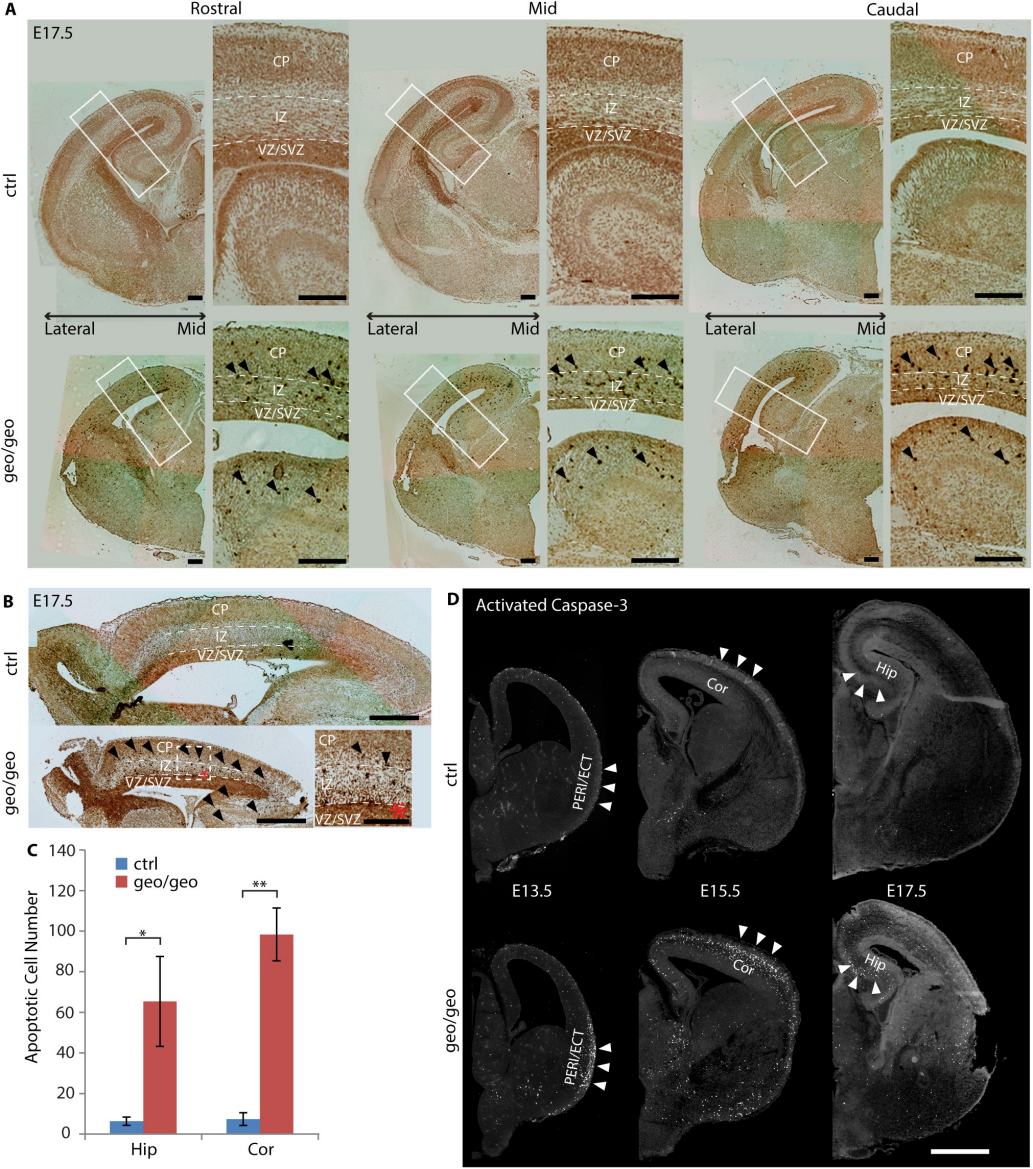
Loss of BIG1 causes neuronal apoptosis. (A-B) TUNEL assay using coronal sections (A, scale bars, 200 μm) and sagittal sections (B, scale bars, 500 μm; *insets, 100 μm) showing apoptotic cells from mid to lateral and rostral to caudal regions of the neocortex at E17.5. n = 3 per group. (C) The numbers of apoptotic cells in the hippocampus (*p = 0.03) and neocortex (**p = 0.002) in *Arfgef1*^*geo/geo*^ brains were significantly greater than in the control brains. Student’s T-test was used to compare between groups. Data are shown as the mean ± SD. n = 3 per group. (D) Immunostaining of activated Caspase-3 showing the progression of the apoptotic population (white arrowheads) from the piriform cortex at E13.5 to neocortex at E15.5 and then into hippocampus at E17.5 in *Arfgef1*^*geo/geo*^ brains. Scale bars, 200 μm. n = 4 per group for each time point. Cor, neocortex; CP, cortical plate; Hip, hippocampus; IZ, intermediate zone; VZ/SVZ, ventricular zone and subventricular zone; PERI/ECT, perirhinal and ectorhinal areas.

Another possibility for the significant reductions in DAPI^+^ cell numbers and the cortical thickness at E17.5 might be apoptosis. Therefore, we checked the *Arfgef1*^*geo/geo*^ brains for apoptosis using TUNEL assay. At E17.5, TUNEL staining of both coronal (Fig 3A) and sagittal sections (Fig 3B) showed that the numbers of apoptotic cells in the hippocampus and neocortex of *Arfgef1*^*geo/geo*^ brains were significantly increased compared to those in control brains (Fig 3C). This increase in apoptosis was observed throughout the neocortex (Fig 3A and 3B), suggesting that the cell loss by apoptosis is a reason for the reduction of neocortical thickness in *Arfgef1*^*geo/geo*^ brains. The apoptotic cells were located outside the VZ/SVZ area in *Arfgef1*^*geo/geo*^ brain sections (Fig 3A and 3B). This was consistent with the earlier finding that NPCs, mainly localized in the VZ/SVZ, were not affected by the loss of BIG1. Instead, the postmitotic neurons are likely to be affected.

Because the reduction of total DAPI^+^ cell numbers also occurred before E17.5, we compared the E13.5, E15.5 and E17.5 brains in search for the peak time point of apoptosis in the neocortex. We found that the apoptosis in the neocortex was most prominent at E15.5 (Fig 3D). Interestingly, the distribution of apoptotic cells gradually progressed from the perirhinal (PERI) and ectorhinal (ECT) areas (E13.5) into the neocortex (E15.5) and then finally into the hippocampus (E17.5) as brain development continued (Fig 3D, white arrowheads). This progression is very similar to the spatial-temporal migration pattern of interneurons arising from the ganglionic eminence (GE) [27, 28].

### Loss of BIG1 did not cause death or migration defect of interneurons

Interneurons can be divided into three major groups based on their location of origin, that is, the dorsal medial ganglionic eminence (dMGE), medial ganglionic eminence (MGE) or caudal ganglionic eminence (CGE) [3]. The pattern of apoptosis progression led us to investigate which interneurons corresponded to the apoptotic population. Calbindin and Prox1 are the markers for interneurons arising from the MGE and CGE, respectively [29, 30]. Double staining for Prox1 and activated Caspase-3 at E15.5 showed no colocalization of these two markers in *Arfgef1*^*geo/geo*^ brains (S1A Fig). We obtained the same result when we double-stained for Calbindin and activated Caspase-3 in *Arfgef1*^*geo/geo*^ brains (S1B Fig). As another major group of interneurons, arising from the dMGE, which expressed Somatostatin (SST), mainly proliferate after postnatal week 1 [31], the numbers of SST^+^ interneurons at E15.5 and E17.5 were too small for the amount of apoptotic cells during this period. Based on these results, interneurons are unlikely to be the main contributor to the apoptotic population.

On the other hand, similar to studies from other groups using wild-type brains [27, 32], both Prox1^+^ and Calbindin^+^ interneurons were found migrating in the neocortex and reaching their destination in the hippocampus at E15.5 and E17.5, respectively (white arrowheads) (S1A and S1B Fig). This indicated that tangential migration of interneurons was not affected by the loss of BIG1.

### Loss of BIG1 caused apoptosis and accumulation of excitatory neurons in IZ

**Fig 4:**
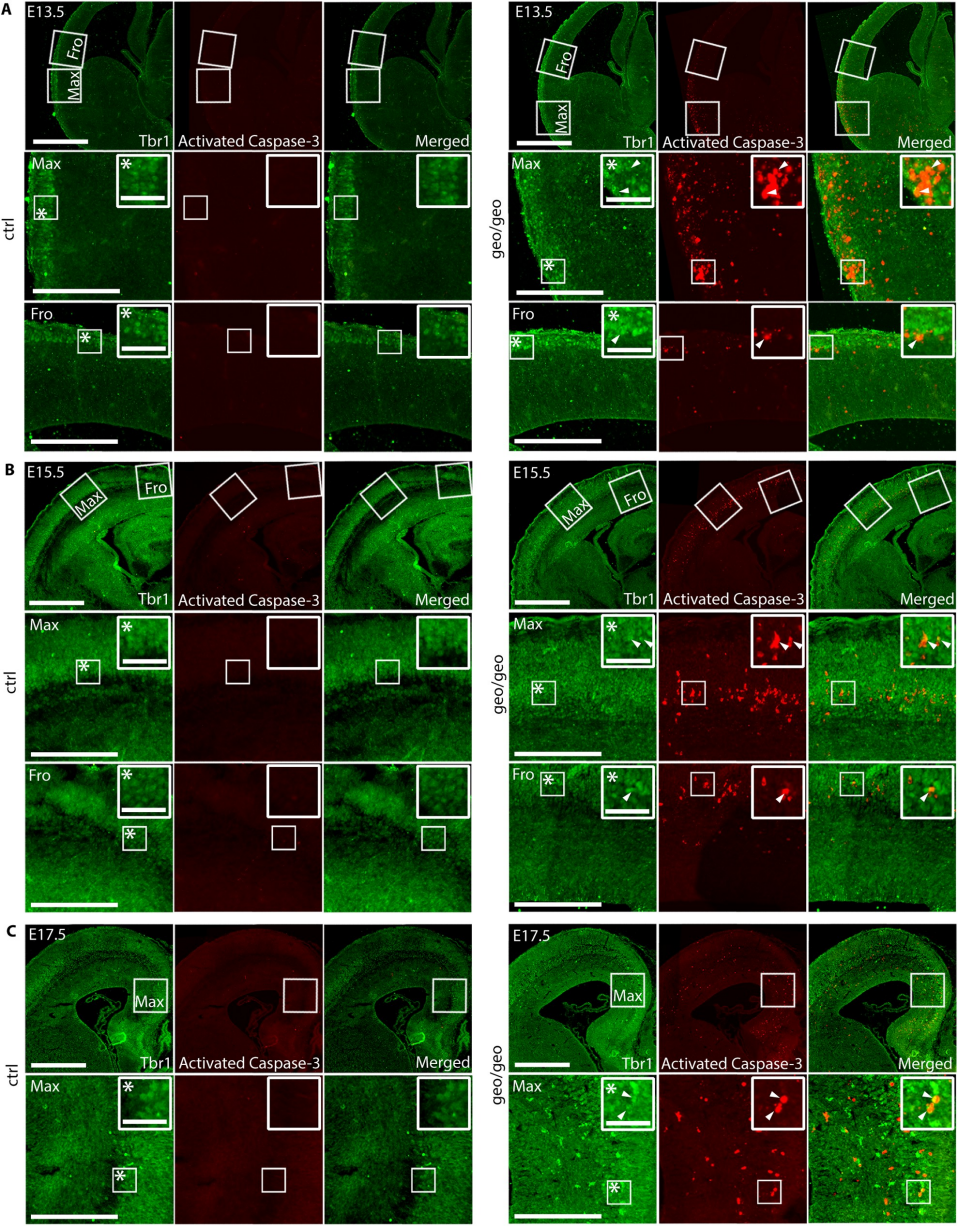
Loss of BIG1 causes cell death of excitatory neurons. (A-C), Activated Caspase-3 colocalized with Tbr1 (white arrowheads) at (A) E13.5, (B) E15.5 and (C) E17.5 in *Arfgef1*^*geo/geo*^ brains. Scale bars, 200 μm; *insets, 50 μm. n = 3 per group. Fro, apoptosis front; Max, maximum apoptosis area.

Since BIG1 did not appear to function in NPCs and the development of interneurons, and our result showed that the apoptotic cells localized around the IZ-CP boundary (Fig 3A), we focused on postmitotic DL excitatory neurons. When we stained the neocortex at E13.5 (Fig 4A), E15.5 (Fig 4B) and E17.5 (Fig 4C), against activated Caspase-3 and Tbr1, a marker of postmitotic DL excitatory neurons, *Arfgef1*^*geo/geo*^ Tbr1^+^ DL neurons were found to colocalize with activated Caspase-3 (Fig 4A–4C, white arrowheads) in a spatial- and temporal-dependent manner. The percentage of colocalization relative to all Caspase-3^+^ cells at E13.5 was 33.97% ±0.91; at E15.5 was 73.65% ±15.31; at E17.5 was 30.13% ±1.63. We also found the colocalization of Caspase-3 with another DL excitatory neuron marker, Ctip2 (S2A–S2C Fig). The percentage of colocalization, at E13.5 is 34.72% ±13.75; at E15.5 is 42.79% ±21.76; at E17.5 is 46.59% ±7.65. This pattern was overlapping with the spatial-temporal migration pattern of interneurons at E13.5, E15.5 and E17.5. This suggested two things. First, this showed that the apoptosis of DL neurons contributed to the cell loss in the *Arfgef1*^*geo/geo*^ neocortex. Second, the spatial-temporal migration of interneurons might have induced the apoptosis of DL neurons in the *Arfgef1*^*geo/geo*^ neocortex.

Excitatory neurons arise from the NPCs in the VZ/SVZ of the neocortex adjacent to the lateral ventricles. These postmitotic neurons then migrate radially into the cortical plate (CP) through the intermediate zone (IZ) and form the DL and UL of the neocortex. The DL neurons differentiate earlier in development. The UL neurons differentiate later and migrate over the DL neurons to form new layers above them [33, 34].

**Fig 5:**
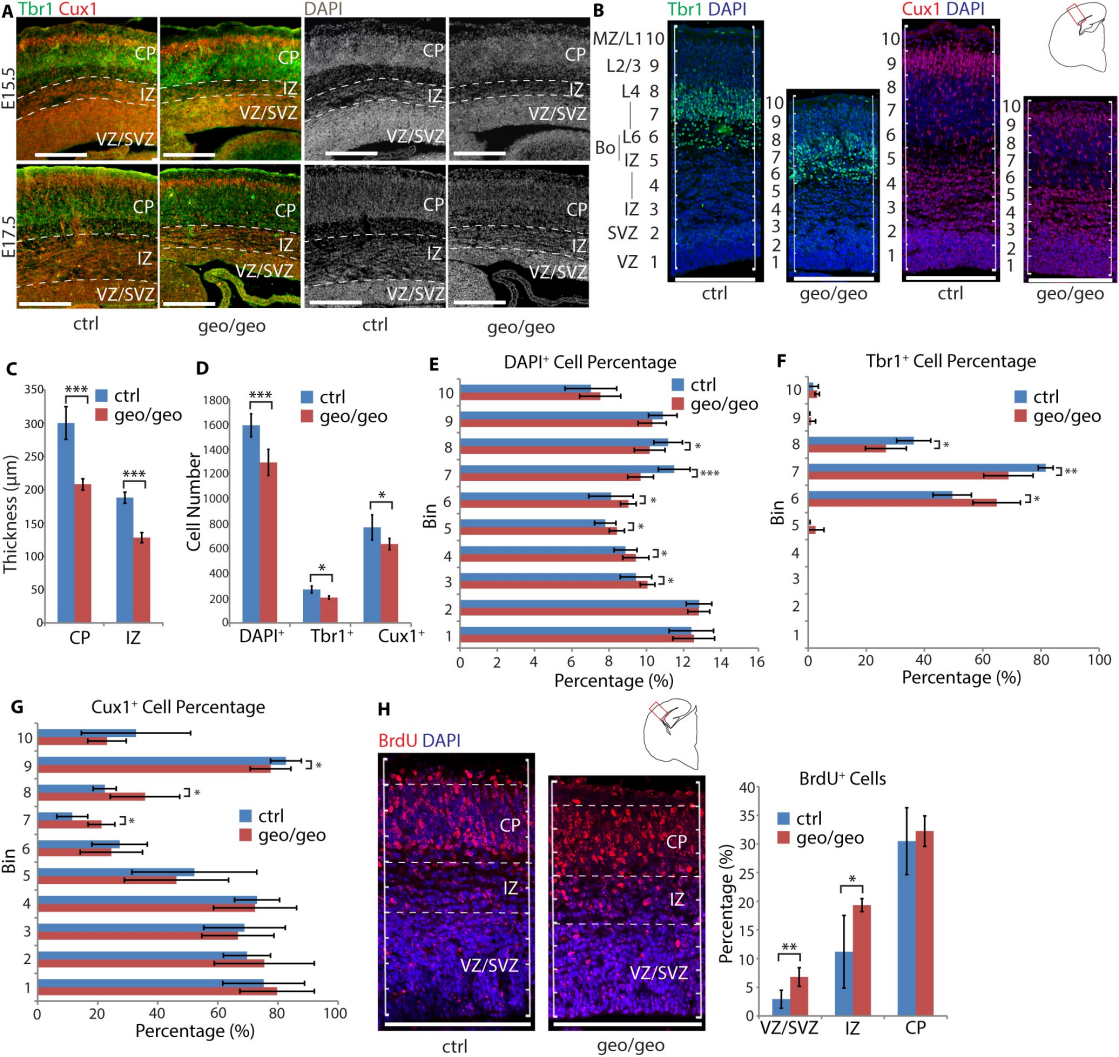
Loss of BIG1 causes accumulation of excitatory neurons before destination layers. (A) The layering order of UL neurons (Cux1^+^) and DL neurons (Tbr1^+^) was maintained in *Arfgef1*^*geo/geo*^ but the IZ thickness reduced at E17.5. Scale bars, 200 μm. n = 3 per group. (B) At E17.5, the numbers of Tbr1^+^, Cux1^+^ and DAPI^+^ cells within a 200 μm cortical width starting from the edge of the lateral ventricle were counted. Scale bars, 200 μm. n = 4 per group. (C) The thickness of the CP (***p = 0.0003) and IZ (***p = 1.25E-06) in *Arfgef1*^*geo/geo*^ brains were significantly reduced. n = 5 per group. (D) In *Arfgef1*^*geo/geo*^ neocortex, the total Tbr1^+^ (*p = 0.02), Cux1^+^ (*p = 0.04) and DAPI^+^ (***p = 0.0007) cells per 200 μm cortical width significantly reduced compared to controls. Tbr1^+^, n = 4 per group; Cux1^+^, n = 4 per group; DAPI^+^, n = 8 per group. (E) In *Arfgef1*^*geo/geo*^ neocortex, DAPI^+^ cells accumulated in Bin 3 (*p = 0.02), Bin 4 (*p = 0.04), Bin 5 (*p = 0.03), Bin 6 (*p = 0.03) at the expense of DAPI^+^ cells in Bin 7 (***p<0.0001), and Bin 8 (*p = 0.01). n = 8 per group. (F) In the *Arfgef1*^*geo/geo*^ neocortex, Tbr1^+^ cells accumulated in Bin 6 (*p = 0.01) and at the expense of Tbr1^+^ cells in Bin 7 (**p = 0.004) and Bin 8 (*p = 0.03). n = 4 per group. (G) The Cux1^+^ cells accumulated in Bin 7 (*p = 0.01) and Bin 8 (*p = 0.03) at the expense of Cux1^+^ cells in Bin 9 (*p = 0.02). n = 4 per group. (H) Pregnant dams at E12.5 were injected with BrdU and the migration of cells in neocortex was quantified at E15.5. The percentage of BrdU^+^ cells in the VZ/SVZ (**p = 0.007) and IZ (*p = 0.04) significantly increased in *Arfgef1*^*geo/geo*^ compared to control brains. However, the percentage of BrdU^+^ cells in CP (p = 0.31) did not decrease. *Arfgef1*^*geo/geo*^, n = 2; controls, n = 4. For all bar graphs, Student’s T-test was used for comparisons between groups. Data are shown as the mean ± SD. Bo, IZ-CP boundary; CP, cortical plate; IZ, intermediate zone; L1-L6, layers 1–6, MZ, marginal zone; VZ/SVZ, ventricular zone and subventricular zone.

Since the Tbr1^+^ DL neurons were found to be involved in the observed apoptosis, we first verified the order of layers in the neocortex at different time points. At E15.5 and E17.5, Cux1^+^ UL neurons remained above Tbr1^+^ DL neurons in the *Arfgef1*^*geo/geo*^ neocortex (Fig 5A). This indicated that the layering order was maintained throughout development. However, DAPI staining in the *Arfgef1*^*geo/geo*^ neocortex showed that the CP-IZ boundary was less clear than in the control neocortex at these time points (Fig 5A). The CP-IZ boundary was the area where apoptotic cells were most abundant (Figs 3A and 4B). This suggested that the loss of postmitotic neurons through apoptosis also disrupted the subplate (SP) between the CP and the IZ. Further, the thickness of the CP and IZ in *Arfgef1*^*geo/geo*^ brains was also significantly reduced (Fig 5C), while the density of cells in the IZ was observed to be higher than in control mice, especially at E17.5 (Fig 5A). This suggested an accumulation of differentiated postmitotic neurons within the IZ of *Arfgef1*^*geo/geo*^ brains. Across the area evaluated, compared to the control neocortex, the *Arfgef1*^*geo/geo*^ neocortex exhibited reductions in the total numbers of DAPI^+^ cells, Tbr1^+^ DL neurons and Cux1^+^ UL neurons (Fig 5D).

**Fig 6:**
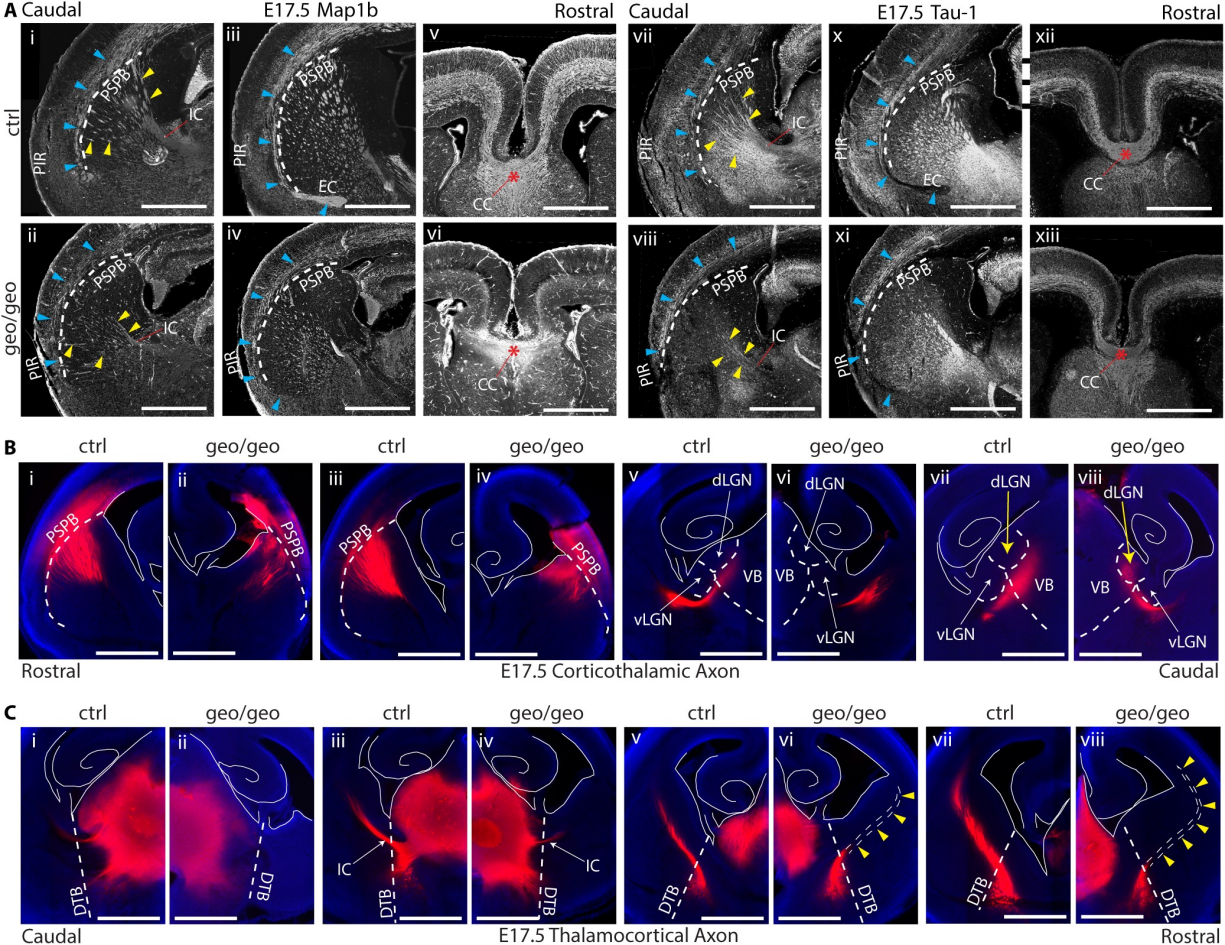
Loss of BIG1 causes defects in axonal projection, axonal pathfinding and axonal extension. (A) At E17.5, there are differences in the projecting direction of cortical axons (blue arrowheads) and in the numbers of axon fascicles passing the PSPB (yellow arrowheads) in *Arfgef1*^*geo/geo*^ brains compared to controls. The CC (red asterisks) in *Arfgef1*^*geo/geo*^ forebrain has no difference compared to the control. Scale bars, 500 μm. n = 3 per group. (B) DiI tracing of shows a pathfinding defect of corticothalamic axons in *Arfgef1*^*geo/geo*^ brains. Scale bars, 500 μm. n = 3 per group. (C) DiI tracing of thalamocortical axons shows an axonal extension defect in *Arfgef1*^*geo/geo*^ thalamic neurons. Scale bars, 500 μm. n = 3 per group. PSPB, pallial-subpallial boundary; CC, corpus callosum; DTB, diencephalon-telencephalon boundary; IC, internal capsule; vLGN, ventral lateral geniculate nucleus; VB, ventrobasal complex; dLGN, dorsal lateral geniculate nucleus.

To evaluate whether UL or DL neurons had ectopic accumulation due to loss of BIG1, 200 μm widths of cortical areas in E17.5 brains were separated into 10 bins, and the numbers of DAPI^+^, Tbr1^+^, and Cux1^+^ cells in each bin were counted (Fig 5B). In the *Arfgef1*^*geo/geo*^ neocortex, more DAPI^+^ cells were accumulated in Bins 3–6 (IZ and IZ-CP boundary). There were fewer cells in Bin 7 and Bin 8 (layer 4 to layer 6) (Fig 5E). This confirmed the observation in Fig 6*A* that the density of DAPI^+^ cells was higher in the IZ of the *Arfgef1*^*geo/geo*^ neocortex. For Tbr1^+^ DL neurons (Fig 5F), significantly larger numbers of these cells accumulated in Bin 6 (layer 6 and IZ-CP boundary). In turn, fewer Tbr1^+^ DL neurons reached their destination in Bin 7 and Bin 8 (layer 4 to layer 6). Next, significantly larger numbers of Cux1^+^ UL neurons (Fig 5G) accumulated in Bin 7 and Bin 8 (layer 4 to layer 6) and fewer cells reached their destinations in Bin 9 (layer 2 to layer 3). Collectively, differentiated postmitotic Tbr1^+^ DL and Cux1^+^ UL neurons were found to accumulate one or two bins below their normal destinations. These results in E17.5 neocortex suggest the possibility of radial neuron migration defect and it was unclear whether these accumulations were cell autonomous or non-autonomous.

To verify this, we performed a BrdU pulse-chase assay to trace the neuron migration. BrdU is an artificial analog of thymidine that can be incorporated into the DNA during mitosis at S-phase [16]. We exposed E12.5 embryos to BrdU and examined the radial neuron migration after 3 days at E15.5, the developing time-point where the apoptosis is most prominent in the *Arfgef1*^*geo/geo*^ neocortex. In *Arfgef1*^*geo/geo*^ neocortex, the BrdU^+^ cell percentages in VZ/SVZ and IZ increased compared to controls (Fig 5H). However, we did not find decrement of the BrdU^+^ cell percentage in CP (Fig 5H). This suggested that the neurons could migrate normally into the CP before the apoptosis progress into the neocortex at E15.5 (Fig 3D). The migration defect found at E15.5 and the accumulation of Tbr1^+^ and Cux1^+^ neurons before their destination layers at E17.5 were most likely non-cell autonomous. High number of apoptosis cells in the IZ and IZ-CP boundary might have blocked the neuron migration path.

### Loss of BIG1 caused axonal connectivity defects

The axons of Cux1^+^ UL neurons connect to the contralateral hemisphere through the corpus callosum (CC) [7, 35]. The loss of Cux1^+^ UL neurons is likely to cause errors in the projections of commissural axons [36]. Meanwhile, postmitotic Tbr1^+^ DL neurons extend axons into the IZ in the neocortex. These axons then extend toward specific domain of the ipsilateral thalamus [37]. The loss of Tbr1^+^ DL neurons gives rise to errors in ipsilateral thalamocortical and corticothalamic axonal projection [38]. Therefore, if massive apoptosis reduced the numbers of post-mitotic excitatory neurons in the *Arfgef1*^*geo/geo*^ neocortex, the projections of these axons might be affected.

To verify this possibility, E15.5 and E17.5 brains were stained for the axonal markers Tau-1 and Map1b. At E15.5, no obvious defect in axonal extension was found in *Arfgef1*^*geo/geo*^ (S3A Fig). However, at E17.5 (Fig 6A), in the *Arfgef1*^*geo/geo*^ brains, axon fascicles were found to extend toward the piriform (PIR) area instead of curving inward to form the external capsule (EC) as in the control brains (Fig 6Ai-iv, vii-xi, blue arrowheads). There were fewer axon fascicles passing the pallial-subpallial boundary (PSPB) and extending toward the internal capsule (IC) (Fig 6Ai-ii, vii-viii, yellow arrowheads) in the *Arfgef1*^*geo/geo*^ brains. In contrast, the commissural axons can project into the contralateral hemisphere through the corpus callosum (CC) (Fig 6Av-vi, xii-xiii, red asterisks; S4B Fig, yellow arrowheads), although the CC thickness relative to brain height or width is thinner in *Arfgef1*^*geo/geo*^ brains (S4A Fig), based on quantification from the results in Fig 6A. This result suggested a selective effect on the axonal projection of DL neurons from the neocortex to thalamus.

Meanwhile, various studies have suggested that axonal projections from the thalamus to cortex (thalamocortical axons) require axonal projections from the cortex to thalamus (corticothalamic axons) for correct invasion and pathfinding into the neocortex [39, 40, 41, 42]. DiI, a lipophilic dye, can diffuse in the lipid bilayers to stain the entire plasma membrane. Small crystals of DiI were placed in the primary somatosensory cortex (S1) and in the ventrobasal thalamus (VB) at E17.5 to study the corticothalamic and thalamocortical axons, respectively.

In the *Arfgef1*^*geo/geo*^ brain, S1 corticothalamic axons extended into the thalamus (Fig 6B). However, these axon fascicles from the *Arfgef1*^*geo/geo*^ neocortex entered the dorsal lateral geniculate nucleus (dLGN) of thalamus instead of the VB area as in the control brains (Fig 6Bvii-viii, yellow arrows). In contrast, VB-originating thalamocortical axons in the *Arfgef1*^*geo/geo*^ thalamus were unable to cross the diencephalic-telencephalic border (DTB) and therefore failed to extend into the neocortex (Fig 6Ci-viii, yellow arrowheads). In addition, the corticothalamic axons only managed to reach the dLGN of the thalamus more caudally (Fig 6Bvi,viii) than in the control brains (Fig 6Bv,vii). Because the *Arfgef1*^*geo/geo*^ brains were smaller (Fig 1G and 1H), this might indicate that these cortical axons extended into the thalamus through a larger trajectory than in the controls.

### Loss of BIG1 delayed but not eliminated axonal elongation in neurons

BIG1 has been known to localize in the Golgi complex [14]. It is involved in the vesicular transport through activation of Arf1 and influences cell polarity [15, 18, 43]. Because the loss of BIG1 appeared to affect the number of axonal fascicles (Fig 6Ai-ii, vii-vii; Fig 6Bi-ii), axonal pathfinding (Fig 6Bvii-viii) and axonal projection (Fig 6Cv-viii), we investigated whether the loss of BIG1 affect the neuronal polarity *in vitro*.

**Fig 7:**
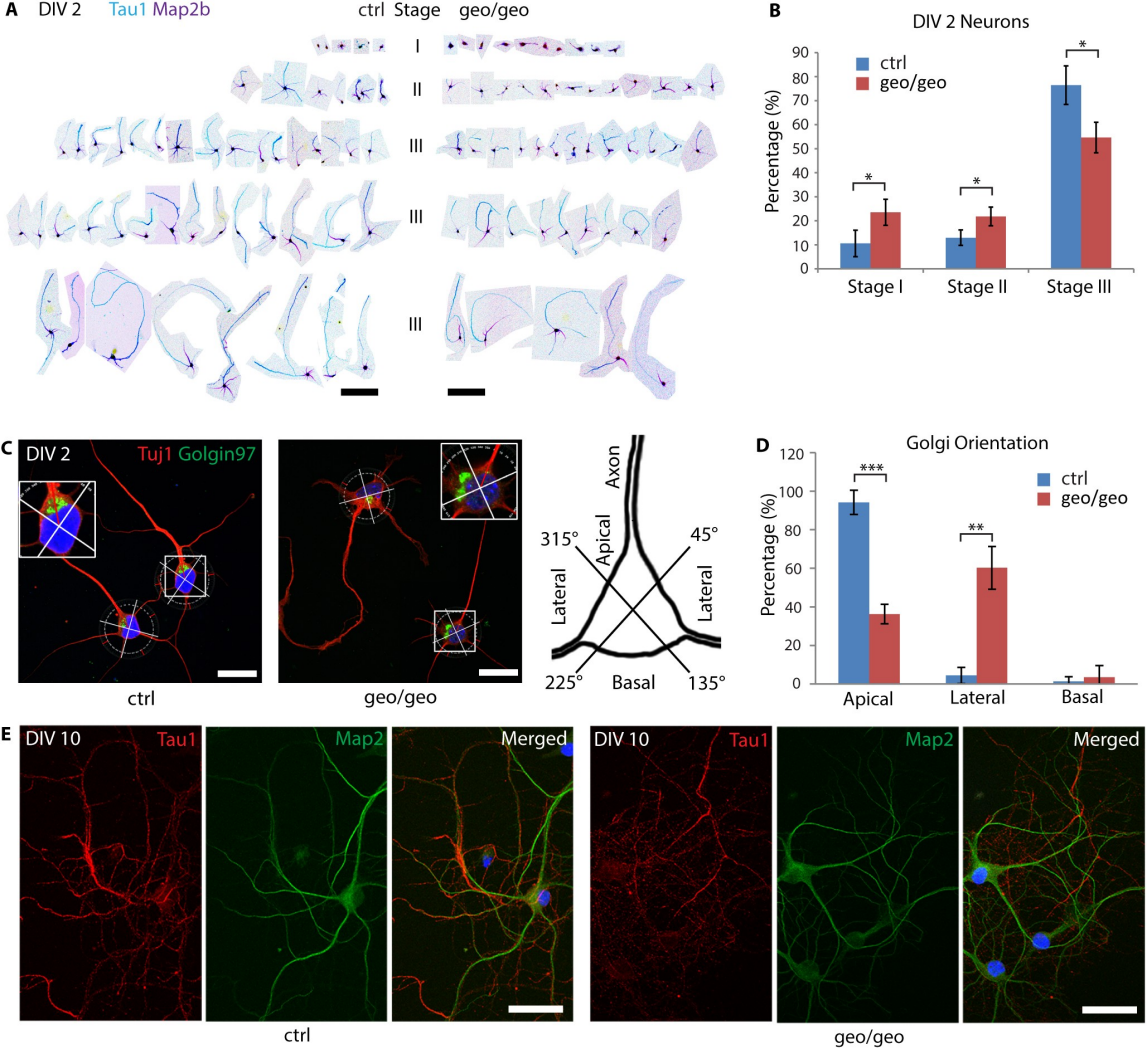
Loss of BIG1 delays early axonal differentiation in neurons. (A-B) In *Arfgef1*^*geo/geo*^ primary hippocampal neuron cultures at DIV 2, the number of stage I (*p = 0.02) and stage II (*p = 0.02) neurons were significantly higher at the expense of stage III neurons (*p = 0.01). Scale bars, 200 μm. n = 50 to 100 neurons per group, from 3 embryos. (C-D), At DIV 2, in *Arfgef1*^*geo/geo*^ neuron cultures, significantly fewer Golgi complexes were localized adjacent to the primary axons (***p = 0.0002) compared to those localized lateral to the primary axons (**p = 0.003). The Golgi complexes localized at basal quadrant were not significantly different between groups (p = 0.31). Scale bars, 20 μm. n = 50 neurons per group from 3 embryos. (E) At DIV 10, the *Arfgef1*^*geo/geo*^ neurons established dendrites and axons as seen in control neurons. Scale bars, 50 μm. n = 50 neurons per group from 3 embryos. For all bar graphs, Student’s T-test was used for comparisons between groups. Data are shown as the mean ± SD.

Because stage III neurons, the youngest neurons with distinguishable axons, are most abundant in day 2 *in vitro* (DIV 2) [44], images of DIV 2 primary hippocampal neurons were collected and aligned (Fig 7*A*) based on developmental stages [45]. The ratios of stage I and stage II neurons to total neurons were significantly higher for the *Arfgef1*^*geo/geo*^ neurons than that for control neurons (Fig 7B), indicating a delay in neuronal polarization. Other parameters, including the length of the longest axon/dendrite, the total length of axons/dendrites, and the number of axon/dendrite branches were similar in the stage III hippocampal neurons between the *Arfgef1*^*geo/geo*^ and control mice (S5A–S5G Fig). The dendrites start to develop from DIV 4, marked by the branching of dendrites [45]. We quantified and compared the percentage of neurons with dendrite branch at DIV 4. No difference was found in between *Arfgef1*^*geo/geo*^ and controls neurons S5H Fig. Therefore, the dendritic development did not seem to be affected by the loss of BIG1.

The determination of the axon has been reported to require the centrosomes, Golgi and endosomes to localize together in close proximity to the neurite that is destined to be an axon [44]. At DIV 2, we observed that the Golgi faced the primary axon in the control neurons [44]. At DIV 7 onward, the Golgi faces the primary dendrite [46]. However, in the *Arfgef1*^*geo/geo*^ neurons at DIV 2, the Golgi complexes were localized significantly more often in the quadrants lateral to the primary axon rather than facing the primary axon (Fig 7C and 7D). At DIV 10, like the control neurons, the *Arfgef1*^*geo/geo*^ neurons developed extensive axons and dendrites (Fig 7E). This suggested that the loss of BIG1 did not completely abolish the neurons’ ability to polarize. Instead, the loss of BIG1 caused the ectopic positioning of the Golgi, which led to a delay in neuronal polarity at early developmental stages.

### Loss of BIG1 induced neuronal hypersensitivity to glutamate

**Fig 8:**
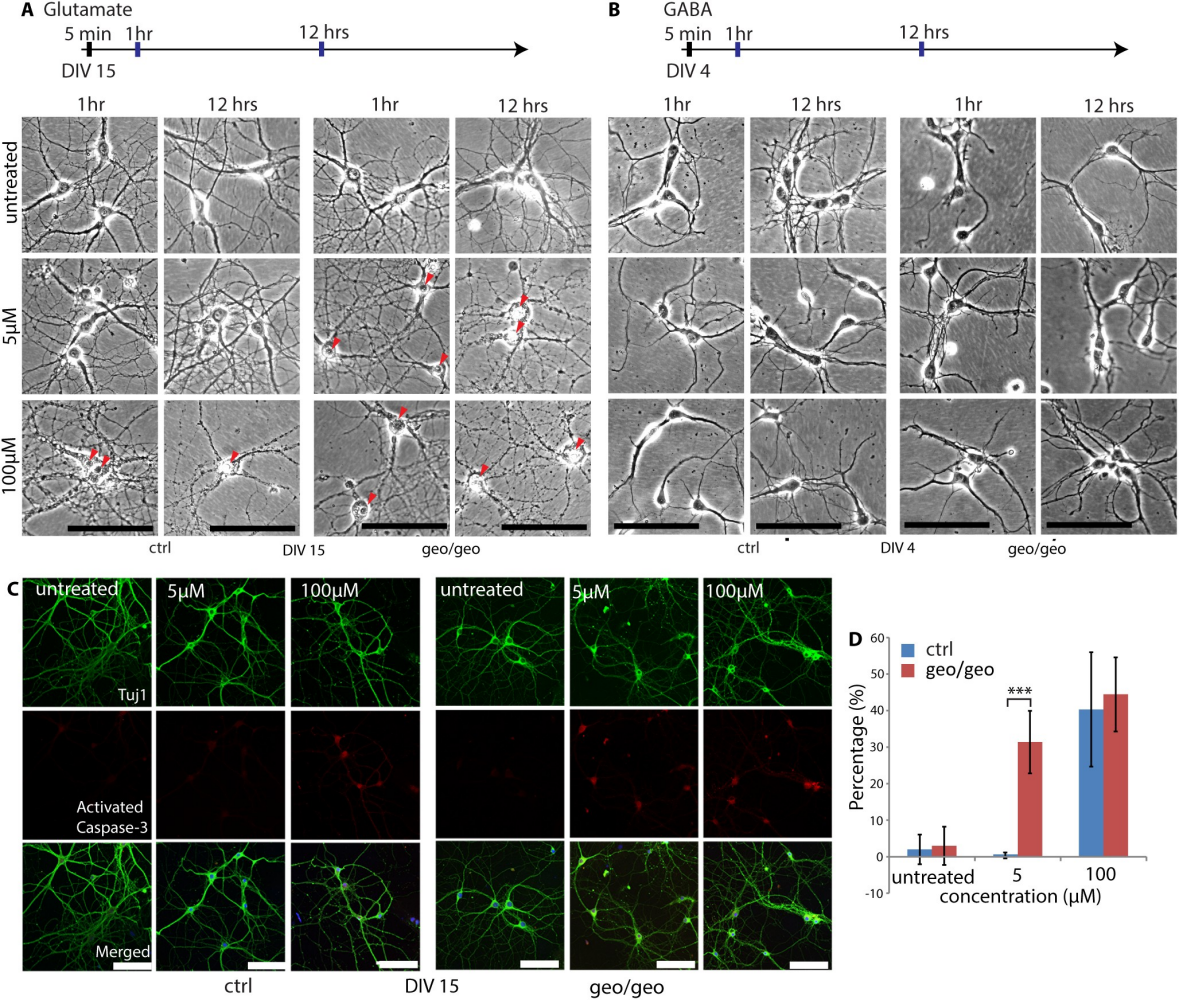
Loss of BIG1 induces hypersensitivity to glutamate in neurons. (A-B) Neurons at DIV 15 and DIV 4 were treated with glutamate (A) and GABA (B) for 5 min and returned to normal culture medium, respectively. The representative images at 1 and 12 hrs after the treatments are shown. At 1 hr and 12 hrs after the 5 μM glutamate treatment, *Arfgef1*^*geo/geo*^ neurons show nuclear shrinkage and membrane blebbing (red arrowheads). The apoptosis phenotypes were not observed in DIV 4 neurons treated with GABA. Scale bars, 100 μm. n = 50 neurons per group from 3 embryos. (C) Neurons were treated with glutamate for 5 min, returned to normal culture medium for 30 min and then stained for Tuj1 and activated Caspase-3. Scale bars, 100 μm. n>50 neurons per group from 2 embryos. (D) Quantification showing that the *Arfgef1*^*geo/geo*^ neurons, treated with 5 μM glutamate, had significantly higher numbers of neurons expressing Activated Caspase-3 compared to the controls at 30 min (untreated, p = 0.26; 5 μM, ***p = 0.00003; 100 μM, p = 0.22). n>50 neurons per group from 2 embryos. Student T-test was used for comparisons between groups. Data are shown as the mean ± SD.

The defect in axonal pathfinding *in vivo* could be due to either extrinsic factors such as secreted neurotransmitters or intrinsic factors, such as apoptosis [42]. Based on our *in vivo* findings, BIG1 appeared to be one of the intrinsic factors causing apoptosis. To determine whether there was involvement of extrinsic factors, the primary hippocampal neurons were examined. Notably, in contrast to neurons *in vivo*, none of the *Arfgef1*^*geo/geo*^ neurons in culture exhibited apoptosis, such as cell body blebbing or chromatin condensation at DIV 2 (Fig 7A), DIV 10 (Fig 7E), and DIV 15 (Fig 8A) in normal culture medium.

Glutamate depolarizes neurons [47]. Stimulation of neurons with a high dose of glutamate induces intracellular accumulation of calcium ions, mitochondrial damage, Caspase-3 activation, and apoptosis [47, 48, 49]. The *Arfgef1*^*geo/geo*^ postmitotic neurons might be hypersensitive to physiological concentrations of the excitatory neurotransmitter glutamate secreted during or after neuronal network formation, thereby leading to apoptosis. Primary hippocampal neurons at DIV 15 were treated with a physiological concentration (5 μM) or a high concentration (100 μM) of glutamate for 5 min in control salt solution (CSS) and then returned to normal culture medium. In the *Arfgef1*^*geo/geo*^ neurons treated with 5 μM glutamate, shrinkage of the nucleus, accompanied by degeneration of neuronal processes was observed beginning at 1 hr after the treatment (Fig 8A, red arrowheads). At 12 hrs, we observed more than half of the *Arfgef1*^*geo/geo*^ neurons exhibited nuclear shrinkage and blebbing of the cell body, hallmarks of apoptosis, accompanied by complete degradation of neuronal processes (Fig 8A, red arrowheads). This indicates that the loss of BIG1 induces glutamate hypersensitivity in postmitotic neurons even at low concentrations. Because another neurotransmitter, GABA, has been known to be excitatory when the neurons are immature [50], we also treated DIV 4 neurons with low and high concentrations of GABA (5 μM and 100 μM). There was no significant cell death being observed in GABA-treated immature neurons up to 12 hours (Fig 8B). To confirm that hypersensitivity to glutamate induced cell death through apoptosis in mature neurons, we stained for activated Caspase-3 at 30 min after low concentration glutamate (5 μM) treatment. There were more *Arfgef1*^*geo/geo*^ neurons than control neurons expressed activated Caspase-3 (Fig 8C and 8D). Therefore, the neuronal apoptosis in *Arfgef1*^*geo/geo*^ brains might be due to the glutamate hypersensitivity occurred only after the postmitotic neurons become mature.

## Discussion

In the current study, the *Arfgef1*^*geo/geo*^ embryos developed until delivery but died soon after birth. Thus, it is hard for us to perform neurological study using these mice. However, there are reduction in the thickness of the neocortex and the hippocampus (Fig 1G and 1H). At later developmental stages, the neocortex and hippocampus consist of three major cell types, NPCs, interneurons, and excitatory neurons. Previous *in vitro* and *in vivo* studies showed that in mice, loss of BIG2, a paralog closely related to BIG1, caused periventricular heterotopia (PH) and impairment of neural migration, which is explained by the BIG2-mediated regulation of Filamin A phosphorylation in NPCs [16, 51]. In the current study, no PH was observed in *Arfgef1*^*geo/geo*^ brains. While we observed a reduction in total DAPI^+^ cells (Fig 2E) and neocortex thickness (Fig 2F) at E17.5 in *Arfgef1*^*geo/geo*^ brains, we did not find statistical differences in the ratio of Pax6^+^:DAPI^+^ cells in the VZ/SVZ (Fig 2D). Next, based on the *Arfgef1* in situ hybridization images from Allen Brain Atlas [52], in neocortex, BIG1 mRNA is expressed in the IZ, CP and hippocampus, but not in the NPC-rich VZ/SVZ area. Collectively, our results suggested that NPCs in the neocortex were not affected by BIG1. This finding was further supported when by our observation that widespread apoptosis occurred only in cells located within the IZ-CP boundary in the *Arfgef1*^*geo/geo*^ neocortex and hippocampus (Fig 3A and 3B), which might be the major reason for the reduced size and thickness of these regions. Overall, our findings suggest the apoptosis of postmitotic neurons in the *Arfgef1*^*geo/geo*^ neocortex.

We next compared the *Arfgef1*^*geo/geo*^ brain at E13.5, E15.5 and E17.5 to identify the period when this apoptosis peaked (Fig 3D). Interestingly, the localization of the apoptotic population at these developmental time points seemed to overlap with the spatial-temporal migration of postmitotic interneurons [27, 28] at respective time point. For example, the apoptosis in the neocortex peaked at E15.5, when the interneurons started to migrate into this area. However, there was no significant colocalization of CGE or MGE interneurons with activated Caspase-3 (S1A and S1B Fig), eliminating the possibility of massive interneuron cell death.

At E17.5, the numbers of Tbr1^+^ and Cux1^+^ neurons were significantly reduced in *Arfgef1*^*geo/geo*^ neocortex (Fig 5G). This supports our finding that the postmitotic neurons were affected by the loss of BIG1. The loss of Tbr1^+^ DL neurons was accompanied by the reduction in Cux1^+^ UL neurons. The reduction in the number of Cux1^+^ UL neurons could be explained by the dependency of the number of UL neurons on that of DL neurons through derepression of Fezf2 by Foxg1 [53]. In the current study, when the number of Tbr1^+^ DL neurons decreased through apoptosis in *Arfgef1*^*geo/geo*^ neocortex, the differentiation of Cux1^+^ UL neurons also decreased based on this dependency, resulting in the thinner neocortex in *Arfgef1*^*geo/geo*^ embryos. Because the upper layer neurons send commissural axons through CC [36], this dependency could also explain the thickness reduction in CC at E17.5 (Fig 6Axii, xiii; S4A Fig). The apoptotic population was mainly localized within the IZ-CP boundary (Fig 3A), which was also overlapping with DL neurons, but not prominent in the UL neurons in the neocortex. Although we could not eliminate the possibility that the apoptosis might occur in migrating UL neurons within IZ, together with the result that the contralateral axonal extension from UL neurons was not affected (Fig 6A), the Cux1^+^ UL populations that had arrived at layer 2–3 were less likely to be further reduced through apoptosis similar to what happened in the DL neurons.

Next, from the BrdU pulse-chase migration assay, unlike the cell-autonomous migration defect found in BIG2 knockout mice, in which the BrdU^+^ neurons in VZ/SVZ and IZ increased at the expense of BrdU^+^ neurons in CP [16], the increment of BrdU^+^ neurons in *Arfgef1*^*geo/geo*^ VZ/SVZ and IZ was not accompanied by the decrement of BrdU^+^ neurons in CP. Because we exposed the embryos with BrdU at E12.5, where the Tbr1^+^ DL neurogenesis begins [54], and checked the neocortex at E15.5, where the apoptosis peaked in the *Arfgef1*^*geo/geo*^ neocortex (Fig 3D), this result suggested that the radial neurons could migrate normally into CP before E15.5. The migration defect we found at E15.5 might be secondary to the progression of apoptosis into the neocortex. Thus, the migration or accumulation defect found after E15.5 in *Arfgef1*^*geo/geo*^ neocortex should be non-cell autonomous.

Tbr1^+^ DL neurons have been found to extend their axons to the thalamus [33]. In the developing neocortex, the primary somatosensory cortex (S1) and ventrobasal thalamus (VB) are innervated at late embryonic stages, whereas the connection between the visual cortex and the dLGN forms postnatally [33, 55]. The timings of these innervations supported our results that there was incorrect S1 –dLGN connectivity instead of the correct S1 –VB connectivity at E17.5 (Fig 6B). The evidences that thalamocortical axon pathfinding requires corticothalamic axon guidance, which induces local changes in attractant or repellent cues that guide the axonal pathfinding [39, 40, 42], further supports our finding that the incorrect connection from corticothalamic projection resulted in the defect in thalamocortical connectivity in the *Arfgef1*^*geo/geo*^ brains (Fig 6C). Apoptosis of DL neurons reduced the number of axons reaching thalamus. Without sufficient local cues, neurons in the thalamus cannot extend their axons into the neocortex. Hence, the neuronal network failed to integrate properly.

In addition to external attractive or repulsive local cues, the defect in neuronal connectivity might be due to the polarization of the neurons themselves. Thus, we evaluated the polarity in primary hippocampal neurons. At DIV 2, the proportion of stage I/II neurons was significantly higher (Fig 7A and 7B), accompanied by the aberrant orientation of the Golgi apparatus (Fig 7C and 7D). However, at DIV 10, all viable neurons had developed axons and dendrites (Fig 7E), suggesting a delay rather than a complete disruption in neuronal polarization, although the latter possibility cannot be excluded. The misorientation of the Golgi at DIV 2 might result in delayed neuronal polarization in the *Arfgef1*^*geo/geo*^ neurons. This finding is supported by the previous finding that HeLa cells treated with BIG1 siRNA displayed misorientation of the Golgi, reduced distribution of actin at the leading edge, and delayed migration in the wound-healing assay [43]. The delayed axonal elongation might result in disruptions in axonal projection and pathfinding and in the connections between various brain regions during development.

We also found that the *Arfgef1*^*geo/geo*^ neurons were hypersensitive to a low dose of glutamate (Fig 8A, 8C and 8D). Glutamate has long been known to involve in the initiation and spread of seizure activity in epilepsy [56]. Apart from elevated level of glutamate, hypersensitive signaling of glutamate in the brain can also lead to epilepsy [57, 58]. Recently, a genetic screening with human as subject had shown that the mutation in *Arfgef1* is one of the possible causes for epileptic encephalopathies [19]. Our study shows that disruption of *Arfgef1* leads to the glutamate hypersensitivity in neurons and also apoptosis of DL neurons in the brain, which might help to explain the mechanism of damage in epilepsy caused by *Arfgef1* mutation. Although we were not able to elucidate the molecular mechanisms of glutamate hypersensitivity in this study, this might involve the regulation of calcium ion concentration in neurons by glutamate receptors [59, 60] and/or by calcium-extrusion proteins [61]. For example, overactivation of the glutamate receptor, NMDAR, enhances influx of calcium ions into neurons, leading to neurotoxicity and is ultimately linked to epilepsy pathogenicity [62, 63]. Meanwhile, in earlier study, NCKX2, a calcium extrusion protein, was found to express abnormally in the hippocampus of epileptic rat [64]. NCKX2 was found to be a cargo of Kif21a in neurons [65]. A previous study showed that Kif21a also interacts with BIG1 in HEK293 cells [66]. Whether or not BIG1 interacts with Kif21a in neurons, as well as whether the loss of BIG1 in neurons affects the binding of Kif21a to NCKX2, remains to be studied.

## Supporting information

S1 Fig Loss of BIG1 does not affect survival or migration of interneurons. (A-B) In *Arfgef1*^*geo/geo*^ brains, activated Caspase-3 does not colocalize with Prox1 at E15.5 (A) or with Calbindin at E17.5 (B). White arrowheads show the Prox1^+^ or Calbindin^+^ cells that migrated into the neocortex and hippocampus. Scale bars, 200 μm; *insets, 50 μm. n = 3 per group. Cor, neocortex; Hip, hippocampus.
(TIF)

S2 Fig Caspase-3 colocalized with Ctip2. (A-C), Activated Caspase-3 colocalized with Ctip2 (white arrowheads) at (A) E13.5, (B) E15.5 and (C) E17.5 in *Arfgef1*^*geo/geo*^ brains. Scale bars, 200 μm; *insets, 20 μm. n = 3 per group. Max, maximum apoptosis area.
(TIF)

S3 Fig Loss of BIG1 does not affect axonal extension at E15.5. (A) The axon extending pattern, visualized by axonal markers Map1b and Tau-1, in the E15.5 *Arfgef1*^*geo/geo*^ brains were similar to the controls. Scale bars, 500 μm. n = 3 per group.
(TIF)

S4 Fig Loss of BIG1 does not affect commissural axon projection. (A) The CC width relative to the brain height (**p = 0.001) or width (***p = 0.0008) is smaller in *Arfgef1*^*geo/geo*^ brains based on the results in Fig 6Axii-xiii. n = 4 per group. (B) At E17.5, dye tracing showed the commissural axons (yellow arrowheads) in *Arfgef1*^*geo/geo*^ brains can reach contralateral brain hemisphere. Scale bars, 500 μm. n = 3 per group. For all bar graphs, Student’s T-test was used for comparisons between groups. Data are shown as the mean ± SD. CC, corpus callosum.
(TIF)

S5 Fig Loss of BIG1 does not affect various parameters of stage III neurons. Various parameters at DIV 2 such as (A) the longest axon length (p = 0.16), (B) the total axon length (p = 0.12), (C) the amount of axon branch (p = 0.36), (D) the longest dendrite length (p = 0.18), (E) the total dendrite length (p = 0.47), (F) the amount of dendrite branch (p = 0.20) and (G) the amount of primary dendrite (p = 0.06) had no significant differences between *Arfgef1*^*geo/geo*^ and control stage III neurons. n>50 neurons per group. (H) DIV 4 *Arfgef1*^*geo/geo*^ neurons can develop long axon (white arrowheads) and dendrites branches (red arrowheads) similar to the control neurons. There was no difference in the percentage of neurons with dendrite branch in *Arfgef1*^*geo/geo*^ and control neurons. n>200 neurons from 4 different embryos per group. Student’s T-test was used for comparisons between groups. Data are shown as the mean ± SD.
(TIF)
