# Supplementary figures and images for: BIG1 is required for the survival of deep layer neurons, neuronal polarity, and the formation of axonal tracts between the thalamus and neocortex in developing brain
Source: PLoS One. 2017 Apr 17;12(4):e0175888. doi: 10.1371/journal.pone.0175888 (PMC5393877; doi:10.1371/journal.pone.0175888)

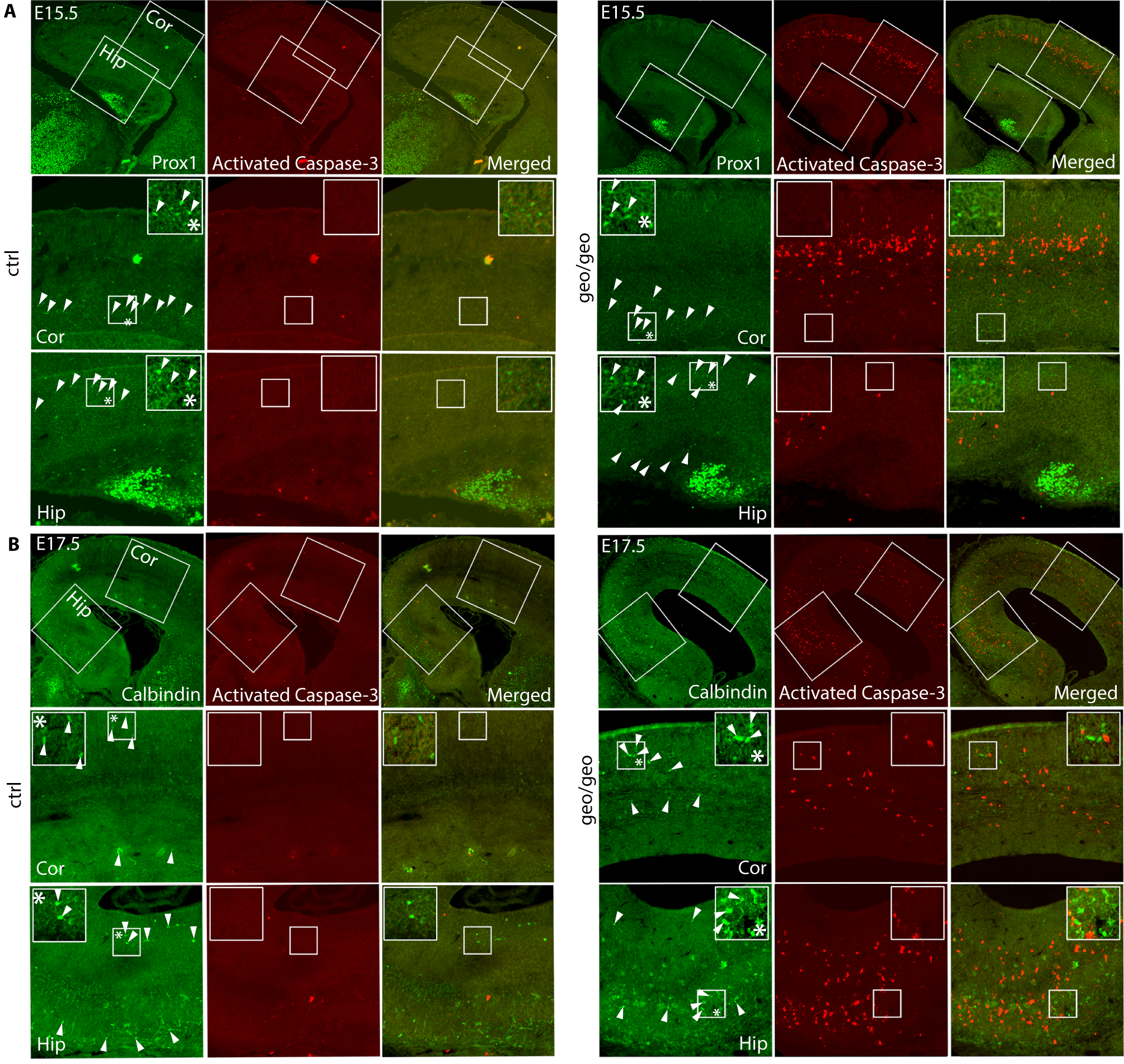

Supplement: S1 Fig — (A-B) In Arfgef1geo/geo brains, activated Caspase-3 does not colocalize with Prox1 at E15.5 (A) or with Calbindin at E17.5 (B). White arrowheads show the Prox1+ or Calbindin+ cells that migrated into the neocortex and hippocampus. Scale bars, 200 μm; *insets, 50 μm. n = 3 per group. Cor, neocortex; Hip, hippocampus. (TIF) [file pone.0175888.s001.tif]

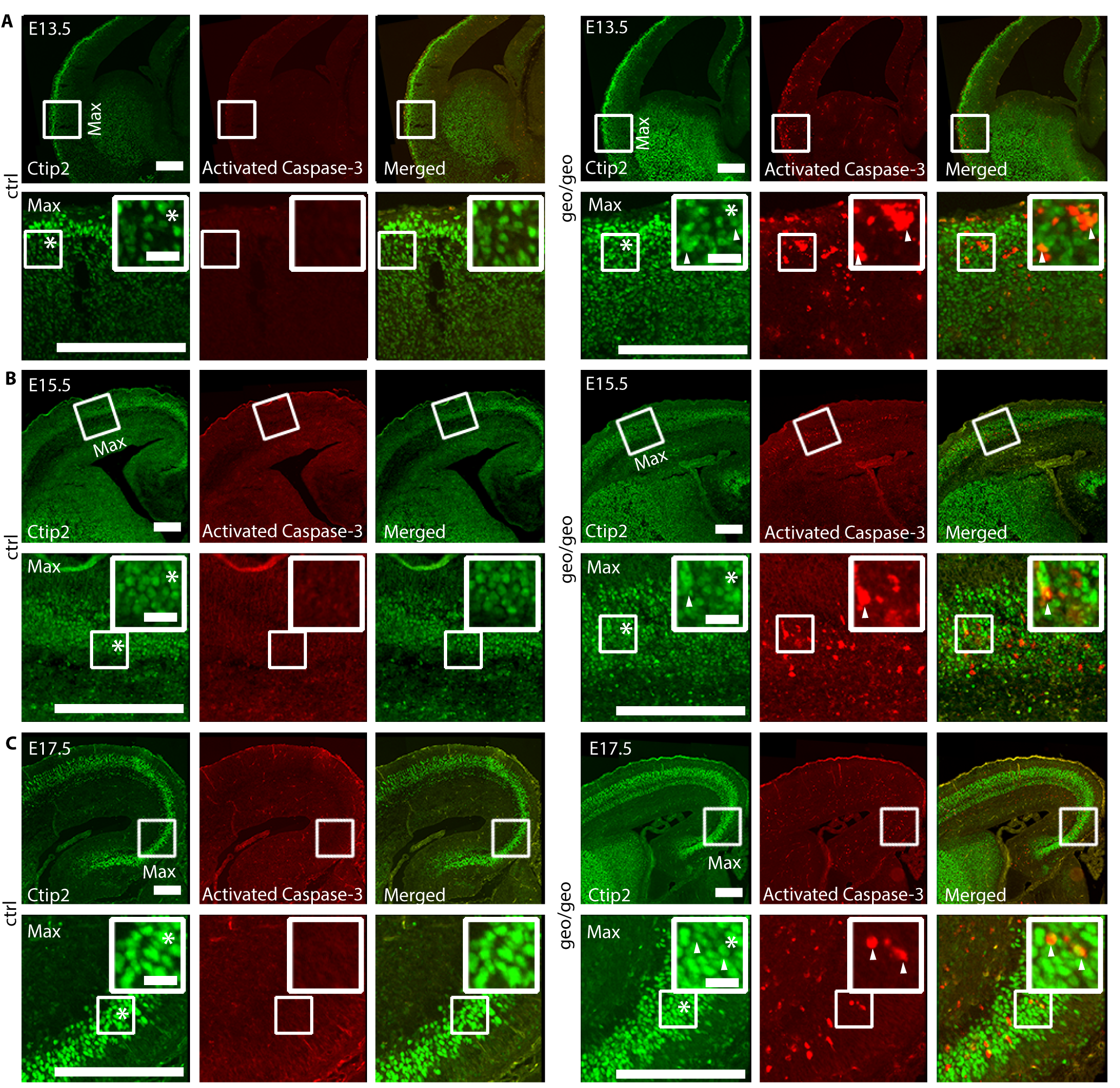

Supplement: S2 Fig — (A-C), Activated Caspase-3 colocalized with Ctip2 (white arrowheads) at (A) E13.5, (B) E15.5 and (C) E17.5 in Arfgef1geo/geo brains. Scale bars, 200 μm; *insets, 20 μm. n = 3 per group. Max, maximum apoptosis area. (TIF) [file pone.0175888.s002.tif]

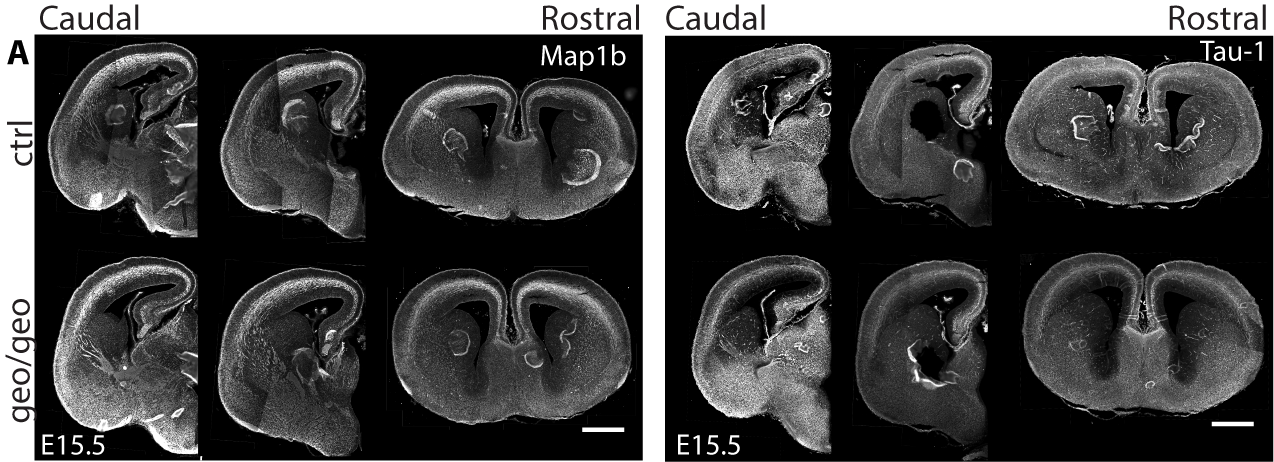

Supplement: S3 Fig — (A) The axon extending pattern, visualized by axonal markers Map1b and Tau-1, in the E15.5 Arfgef1geo/geo brains were similar to the controls. Scale bars, 500 μm. n = 3 per group. (TIF) [file pone.0175888.s003.tif]

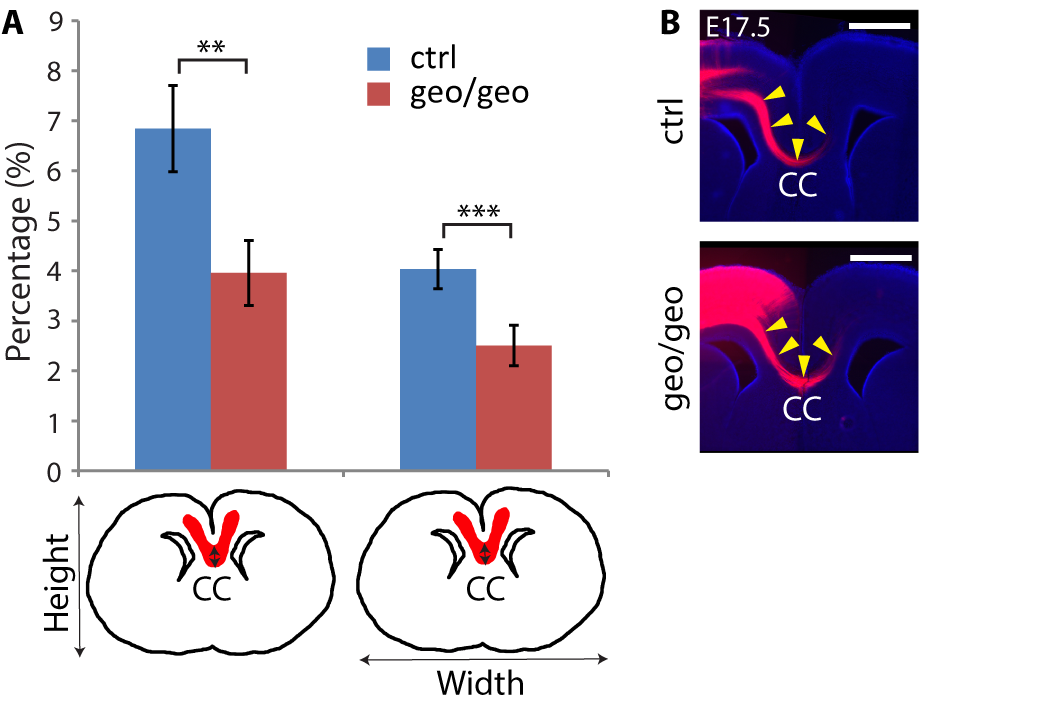

Supplement: S4 Fig — (A) The CC width relative to the brain height (**p = 0.001) or width (***p = 0.0008) is smaller in Arfgef1geo/geo brains based on the results in Fig 6Axii-xiii. n = 4 per group. (B) At E17.5, dye tracing showed the commissural axons (yellow arrowheads) in Arfgef1geo/geo brains can reach contralateral brain hemisphere. Scale bars, 500 μm. n = 3 per group. For all bar graphs, Student’s T-test was used for comparisons between groups. Data are shown as the mean ± SD. CC, corpus callosum. (TIF) [file pone.0175888.s004.tif]

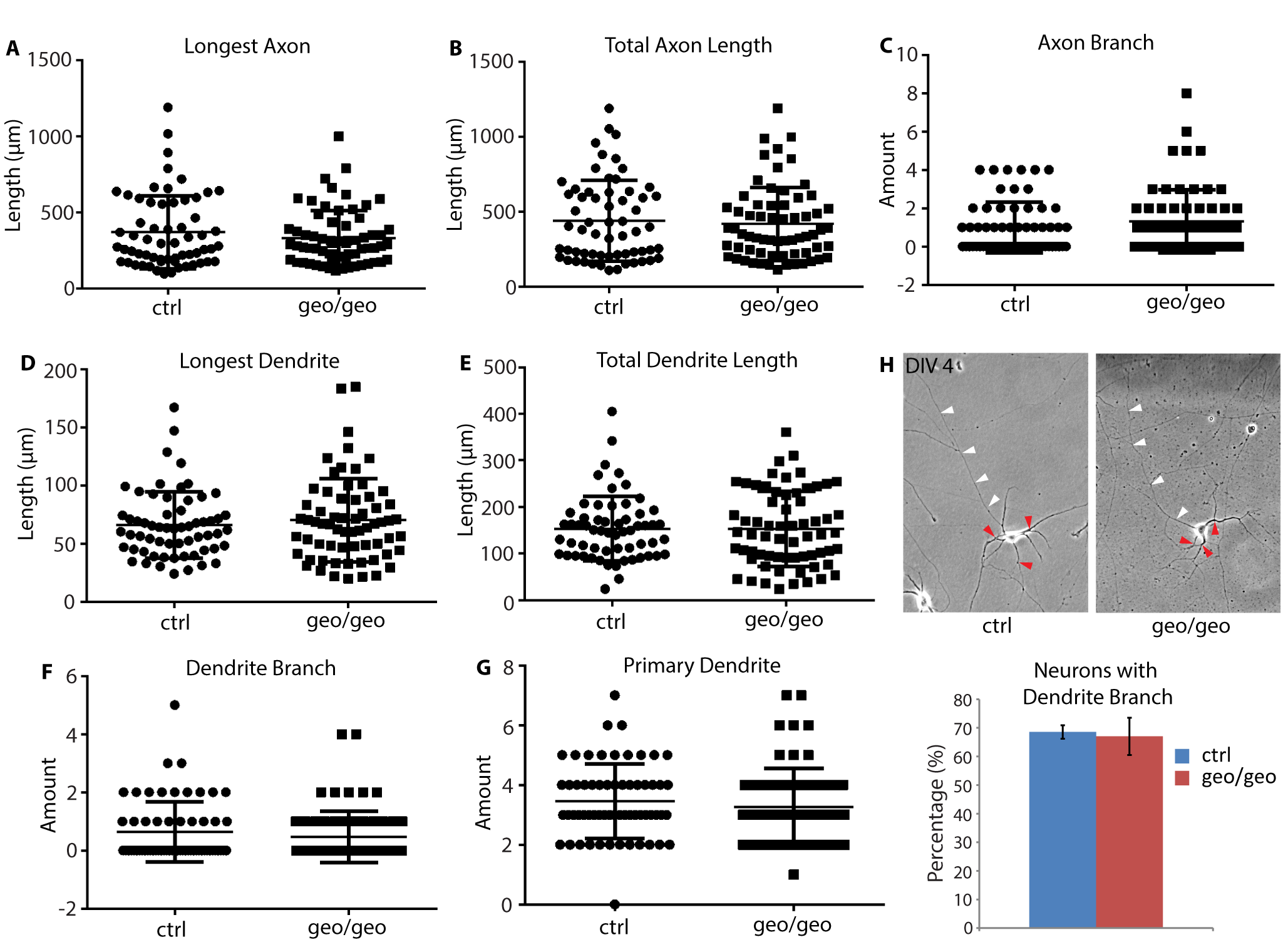

Supplement: S5 Fig — Various parameters at DIV 2 such as (A) the longest axon length (p = 0.16), (B) the total axon length (p = 0.12), (C) the amount of axon branch (p = 0.36), (D) the longest dendrite length (p = 0.18), (E) the total dendrite length (p = 0.47), (F) the amount of dendrite branch (p = 0.20) and (G) the amount of primary dendrite (p = 0.06) had no significant differences between Arfgef1geo/geo and control stage III neurons. n>50 neurons per group. (H) DIV 4 Arfgef1geo/geo neurons can develop long axon (white arrowheads) and dendrites branches (red arrowheads) similar to the control neurons. There was no difference in the percentage of neurons with dendrite branch in Arfgef1geo/geo and control neurons. n>200 neurons from 4 different embryos per group. Student’s T-test was used for comparisons between groups. Data are shown as the mean ± SD. (TIF) [file pone.0175888.s005.tif]
